# Observational evidence of ring current in the magnetosphere of Mercury

**DOI:** 10.1038/s41467-022-28521-3

**Published:** 2022-02-17

**Authors:** J.-T. Zhao, Q.-G. Zong, C. Yue, W.-J. Sun, H. Zhang, X.-Z. Zhou, G. Le, R. Rankin, J. A. Slavin, J. M. Raines, Y. Liu, Y. Wei

**Affiliations:** 1 Institute of Space Physics and Applied Technology, Peking University, Beijing, 100871 China; 2 Polar Research Institute of China, Shanghai, 200136 China; 3 Department of Climate and Space Sciences and Engineering, University of Michigan, Ann Arbor, MI 48109 USA; 4 Geophysical Institute, University of Alaska Fairbanks, Fairbanks, AK 99775 USA; 5 ITM Laboratory, Heliophysics Science Division, NASA Goddard Space Flight Center, Greenbelt, MD 675 USA; 6 Department of Physics, University of Alberta, Edmonton, AB T6G2R3 Canada; 7 Institute of Geology and Geophysics, Chinese Academy of Sciences, Beijing, 100029 China

**Keywords:** Magnetospheric physics, Inner planets

## Abstract

The magnetic gradient and curvature drift of energetic ions can form a longitudinal electric current around a planet known as the ring current, that has been observed in the intrinsic magnetospheres of Earth, Jupiter, and Saturn. However, there is still a lack of observational evidence of ring current in Mercury’s magnetosphere, which has a significantly weaker dipole magnetic field. Under such conditions, charged particles are thought to be efficiently lost through magnetopause shadowing and/or directly impact the planetary surface. Here, we present the observational evidence of Mercury’s ring current by analysing particle measurements from MErcury Surface, Space Environment, GEochemistry, and Ranging (MESSENGER) spacecraft. The ring current is bifurcated because of the dayside off-equatorial magnetic minima. Test-particle simulation with Mercury’s dynamic magnetospheric magnetic field model (KT17 model) validates this morphology. The ring current energy exceeds $5\times {10}^{10}$ J during active times, indicating that magnetic storms may also occur on Mercury.

## Abstract (web summary)

Ring currents have been observed in the magnetospheres of Earth, Jupiter, and Saturn. Here, the authors show observational evidence of Mercury’s ring current that is bifurcated because of the dayside off-equatorial magnetic minima.

## Introduction

The ring current is a magnetospheric electric current mainly carried by ~keV to hundreds of keV ions trapped in a planetary magnetosphere^1–5^. Chapman and Ferraro^1^ first proposed that the ring current, carried by energetic charged particles circling the Earth, causes geomagnetic depressions (i.e., geomagnetic storms)^1^. Frank^6^ confirmed its existence at *L*~3-5 in Earth’s magnetosphere by in situ particle measurements of the OGO 3 satellite. The definition of ring current has decoupled from geomagnetic storms as the exploration of the planetary magnetosphere progressed. A general ring current refers to the longitudinal electric current that results from the drift motion of energetic particles in the investigations of planetary magnetospheres such as Jupiter’s and Saturn’s^2,3^.

Mercury’s magnetosphere was discovered by Mariner-10 in the 1970s^7^. It has an intrinsic dipole field with northwards offset of 0.2 ${{{{{{\rm{R}}}}}}}_{{{{{{\rm{M}}}}}}}$ (Mercury’s Radius, i.e., 2440 km) and a small magnetic moment of $\sim 190\ {{{{{\rm{nT}}}}}}\cdot {{{{{{\rm{R}}}}}}}_{{{{{{\rm{M}}}}}}}^{3}$^8–10^. The subsolar magnetopause is located at ~$1.45\ {{{{{{\rm{R}}}}}}}_{{{{{{\rm{M}}}}}}}$ during normal solar wind conditions^11,12^. During severe solar wind conditions, Mercury’s dayside magnetosphere may even disappear^13,14^. Recent observations of MESSENGER (MErcury Surface, Space ENvironment, GEochemistry, and Ranging) confirmed that Mercury’s magnetosphere resembles Earth’s in many aspects, such as magnetospheric structures (e.g., magnetotail, plasma mantle, and polar cusp), magnetospheric dynamic processes (e.g., flux loading–unloading, substorm dipolarizations), and magnetic structures (e.g., dipolarization fronts and flux ropes)^15–18^. However, in situ measurements to directly demonstrate the existence of Mercury’s ring current are still lacking.

Previous substorm observations have shown that rapid, frequent, and intense ion injection and energization processes occur in Mercury’s magnetotail, which could energetic protons to the inner magnetosphere^19^. However, these energetic particles were considered very hard to be stably trapped due to strong magnetopause shadowing and surface absorption^20,21^. In situ measurements in the magnetotail have revealed quasi-trapped protons (i.e., protons that can only drift for a finite amount of time that is shorter than a complete drift period) with appreciable flux and showed their strong diamagnetic effect during some events^22^. Both global magnetohydrodynamic (MHD) and hybrid (kinetic ions, electron fluid) simulations have also reproduced quasi-trapped particles^22–25^. Whether these energetic particles could drift around Mercury and form a ring current similar to that on Earth is still under debate. In some hybrid and global MHD simulations^26,27^, the drifting ions do not contribute to a notable ring current, especially on the dayside. Meanwhile, the hybrid model results of Exner et al.^28^ demonstrated a remarkable ring current structure only under nominal solar wind dynamic pressure in both nightside and dayside magnetospheres around the magnetic equator (in this manuscript, equator refers to the magnetic equator instead of the geographical equator). For moderate solar wind conditions, the magnetic minimum on the dayside deviates from the magnetic equatorial plane to off-equatorial latitude due to strong solar wind compression, which could result in a bifurcated ring drift shell (i.e., Shabansky orbit^29^). Test particle simulation suggested that 34 keV electrons with specified initial position and pitch angle can completely drift around the planet via Shabansky orbit^30^, which is consistent with the statistical result of low-energy (1–10 keV) or supra-thermal electrons^31–33^. In a case study, Jang et al.^34^ demonstrated the polar cusp trapping of energetic protons under strong solar wind compression based on MESSENGER observations. The above works suggest that the ring current morphology may depend on the dynamic pressure of the solar wind. Apart from the proton, the sodium ion is also considered to contribute to the partial ring current. Hybrid simulations performed by Paral et al.^35^ suggest that the exospheric sodium ions released by photostimulated desorption and solar wind sputtering could form a partial ring current-like density pattern. Furthermore, the latest simulations performed by Yagi et al.^36^ and Exner et al.^28^ demonstrate a complete and intense sodium ring current under the impact of a small solar wind dynamic pressure. Nevertheless, in situ observations and simulations are still required to determine whether energetic protons originating from the magnetotail can complete a full drift orbit and form a ring current, how the protons are distributed and how strong the ring current is.

In this study, we present conclusive evidence of Mercury’s ring current based on MESSENGER’s in situ proton and magnetic field observations. This ring current is bifurcated on the dayside due to the poleward mirror force at the subsolar equator. During active times, the total energy of ring current exceeds 5×$1{0}^{10}$ J, demonstrating the possibility of Mercury’s magnetic storm.

## Results

### Dayside observations

**Fig. 1:**
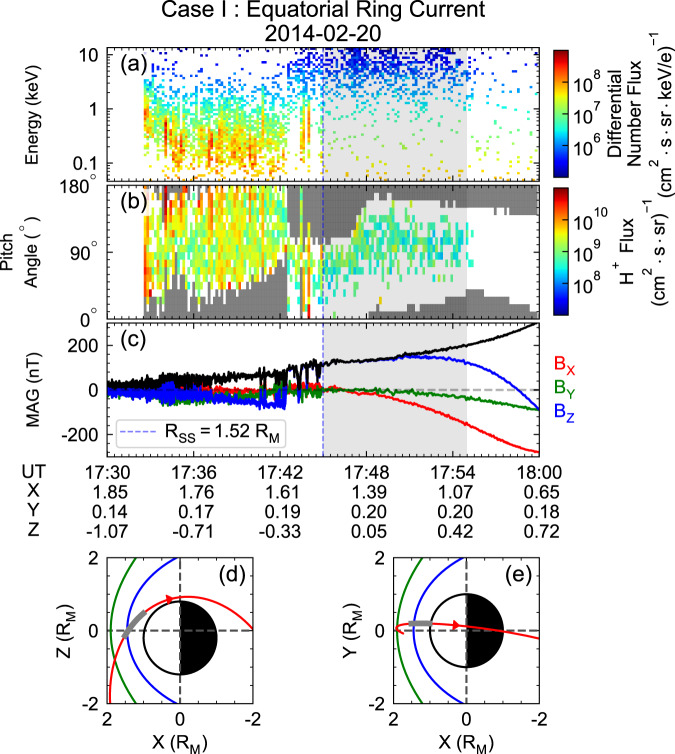
MESSENGER observations during dayside magnetosphere crossing with potential equatorial ring current on Feb 20^th^, 2014. **a** Energy spectrum of the protons. **b** Corresponding pitch angle distribution, with uncovered pitch angle bins noted by grey grids. **c** Components of the magnetic field (red, green, and blue solid lines represent the ${B}_{x},{B}_{y}\ {{{{{\rm{and}}}}}}\ {B}_{z}$ components, respectively) and strength (solid black line). **d**, **e** the trajectory of MESSENGER in XZ, XY planes. The thick shaded areas in **a**–**c** indicate the time periods during which FIPS observed trapped energetic protons. The corresponding spacecraft locations during these intervals are plotted in **d** and **e** as bold grey lines, and the green and blue curves show the modelled dayside bow shock and magnetopause that is obtained from the statistical distribution of observed crossing points^12^, respectively. The white and black half-circles represent the dayside and nightside hemisphere of Mercury, respectively. See Supplementary Fig. 7 for the colour alternative version of this figure.

**Fig. 2:**
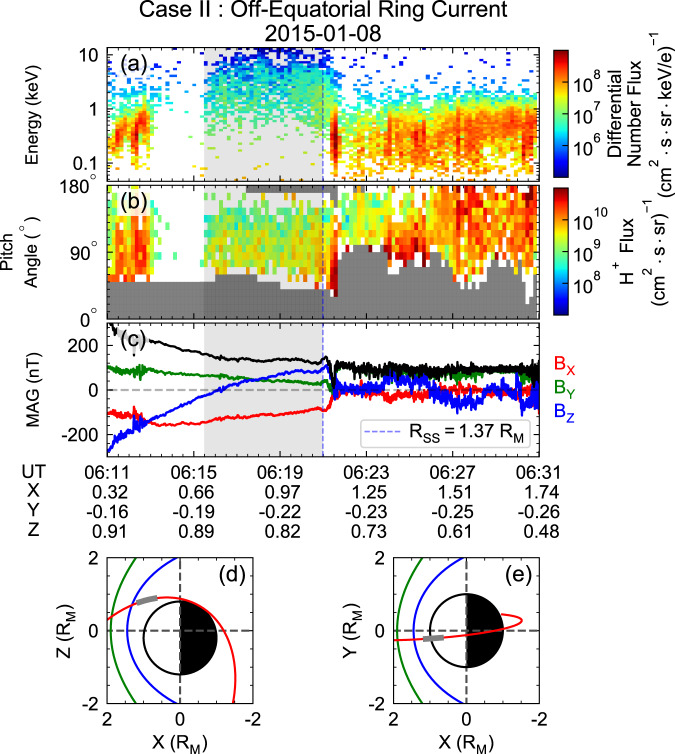
MESSENGER observations during dayside magnetosphere crossing with potential off-equatorial ring current on Jan 8^th^, 2015. **a** Energy spectrum of the protons. **b** Corresponding pitch angle distribution, with uncovered pitch angle bins noted by grey grids. **c** Components of the magnetic field (red, green, and blue solid lines represent the ${B}_{x},{B}_{y}\ {{{{{\rm{and}}}}}}\ {B}_{z}$ components, respectively) and strength (solid black line). **d**, **e** the trajectory of MESSENGER in XZ, XY planes. The thick shaded areas in **a**–**c** indicate the time periods during which FIPS observed trapped energetic protons. The corresponding spacecraft locations during these intervals are plotted in **d**–**e** as bold grey lines, and the green and blue curves show the modelled dayside bow shock and magnetopause that is obtained from the statistical distribution of observed crossing points^12^, respectively. The white and black half-circles represent the dayside and nightside hemisphere of Mercury, respectively. See Supplementary Fig. 8 for the colour alternative version of this figure.

Observations of energetic protons in the dayside magnetosphere are crucial to determine whether energetic protons injected from Mercury’s magnetotail can complete a full drift orbit and form a ring current. This section presents two Mercury’s dayside magnetosphere crossings (Figs. 1 and 2) with potential ring current observed by MESSENGER. The energy spectrum and pitch angle distribution (PAD) measured by the FIPS instrument are presented in the first two rows (Figs. 1a, b and 2a, b), and the magnetic field observations are shown in Panels 1c and 2c. The trajectories of the spacecraft are presented in Figs. 1d, e and 2d, e.

In the first case (2014-02-20), MESSENGER made an inbound magnetopause crossing at 1745 UT. The observations of proton flux (Fig. 1a, b) and magnetic field (Fig. 1c) show abrupt changes when the spacecraft entered the magnetosphere. According to the position (*X* = 1.51 ${{{{{{\rm{R}}}}}}}_{{{{{{\rm{M}}}}}}}$, *Y* = 0.20 ${{{{{{\rm{R}}}}}}}_{{{{{{\rm{M}}}}}}}$, *Z* = $-$0.$13\ {{{{{{\rm{R}}}}}}}_{{{{{{\rm{M}}}}}}}$, dashed blue line in Fig. 1a–c) of the magnetopause, its subsolar standoff distance can be estimated by adopting the following functional form of the Shue model^12,37^:

1 $${R}_{{SS}}=R\cdot \sqrt{\frac{1+X/R}{2}}$$

where R is the radial distance of the magnetopause location. The result of 1.52 ${{{{{{\rm{R}}}}}}}_{{{{{{\rm{M}}}}}}}$ subsolar distance is larger than the average size of 1.45 ${{{{{{\rm{R}}}}}}}_{{{{{{\rm{M}}}}}}}$, indicating that Mercury is under relatively weak solar wind forcing^12^. From 1745 to 1755 UT (grey shaded region marked in Fig. 1a–c and bold grey segment of the red trajectory line in Fig. 1d, e), ~1–13 keV proton fluxes are significantly enhanced near the dayside equatorial plane (*X*~1.0–1.5 ${{{{{{\rm{R}}}}}}}_{{{{{{\rm{M}}}}}}}$), with corresponding pitch angles from ${50}^{\circ }$ to ${130}^{\circ }$. In addition, the magnetic field strength during this time interval was $\sim 160\ {{{{{\rm{nT}}}}}}$, with B_z_ being the dominant component near the magnetic equator, indicating that the spacecraft was near the equator.

The second case (2015-01-08) presents a polar magnetopause crossing with energetic proton flux enhancement, as in Case I. However, the energetic protons were located at high latitudes (${\sim 45}^{\circ }{{{{{\rm{N}}}}}}$) instead of around the equator, as shown in Fig. 2d, e. The estimated subsolar distance of the magnetopause during this crossing is 1.37 ${{{{{{\rm{R}}}}}}}_{{{{{{\rm{M}}}}}}}$ using the same method, indicating a relatively small magnetosphere and intense solar wind dynamic pressure (${p}_{{dyn}}$). It should be noted that the estimation of magnetopause subsolar distance from polar magnetopause crossing may be affected by cusp indentation^38^. This distance will be 1.36 ${{{{{{\rm{R}}}}}}}_{{{{{{\rm{M}}}}}}}$ by adopting the magnetopause model with cusp indentation in Zhong et al.^38^, still indicating intense solar wind forcing. Under such strong compression (${R}_{{SS}}=1.37\ {{{{{{\rm{R}}}}}}}_{{{{{{\rm{M}}}}}}}$), we expect that energetic protons originating from the nightside plasma sheet do not travel through the magnetic equator because of the strong magnetic field of the dayside [one example of a low flux level of energetic protons near the equator under high solar wind dynamic pressure (${R}_{{SS}}=1.33\ {{{{{{\rm{R}}}}}}}_{{{{{{\rm{M}}}}}}}$) is presented in Supplementary Fig. 1. Instead, the bifurcation of drift shells (i.e., Shabansky orbit) would be generated; thus, high fluxes of energetic protons are observed at the high latitude regions, as shown in Fig. 2a^30,39^.

The above observations demonstrate the existence of ~${90}^{\circ }$ pitch angle protons with 1–10 keV energy in Mercury’s dayside magnetosphere during both high and low ${p}_{{dyn}}$. Since localised energizations on the dayside (e.g., magnetic reconnection and centrifugal acceleration in the polar cusp) hardly produce $\sim 10\ {{{{{\rm{keV}}}}}}$ protons, these protons are most likely transported from the magnetotail via magnetic gradient-curvature drift. Such inference is also consistent with the observed ${90}^{\circ }$-dominant pitch angle distribution and $\sim 160\ {{{{{\rm{nT}}}}}}$ ambient magnetic field strength, which are similar to the characteristics of protons and the magnetic field strength in the near-Mercury magnetotail^40^. To validate this contention, we now present the results from a test particle simulation and a statistical data analysis in the following sections.

### Comparison of observations and test particle simulations

We use a test particle simulation with the latest dynamic magnetic field model (KT17) of Mercury to investigate the morphology of Mercury’s ring current^41^. The KT17 model is an empirical magnetic field model based on magnetic field observations from MESSENGER’s ~4000 orbits. It shows good agreement with the observed magnetic field (root mean square residual ≈ 20–30 nT). The KT17 model is currently one of the most accurate magnetic field models and is suitable for our simulation, although it has several limitations, which are listed in the Methods, subsection limitations of KT17 model.

Here, we release a pair of 5 keV protons from (−1.2 ${{{{{{\rm{R}}}}}}}_{{{{{{\rm{M}}}}}}}$, 0 ${{{{{{\rm{R}}}}}}}_{{{{{{\rm{M}}}}}}}$, 0 ${{{{{{\rm{R}}}}}}}_{{{{{{\rm{M}}}}}}}$) in the magnetotail with pitch angles of ${50}^{\circ }$ and ${130}^{\circ }$, corresponding to a magnetic mirror point latitude of $\sim {15}^{\circ }$ and a field strength of ~160 nT. Only gravity and Lorentz forces are considered in our simulation. A convection electric field may also affect the particle trajectory, but it is not considered due to the lack of both in situ electric field measurements and a self-consistent electric field model of Mercury. Particle trajectory tracing for these protons is performed in the static KT17 modelled magnetic field, with the full gyro-orbit considered rather than the motion of the guiding centre (See methods, subsection test particle simulation). The computation is performed using a fourth-order Runge–Kutta method with a time step adjusted to 1/1000 of the gyration period that is updated in real time. The input parameters of the KT17 model, including the heliocentric distance (${r}_{{Hel}}$, in astronomical unit, AU) and the disturbance index (DI)^42^, are set to the average values (i.e., $0.387\ {{{{{\rm{AU}}}}}}$ and 50) for constructing a regular magnetosphere. In such a situation, the subsolar magnetopause distance is $1.41\ {{{{{{\rm{R}}}}}}}_{{{{{{\rm{M}}}}}}}$, which is within the deviation range of previous statistics on the mean magnetopause location.

**Fig. 3:**
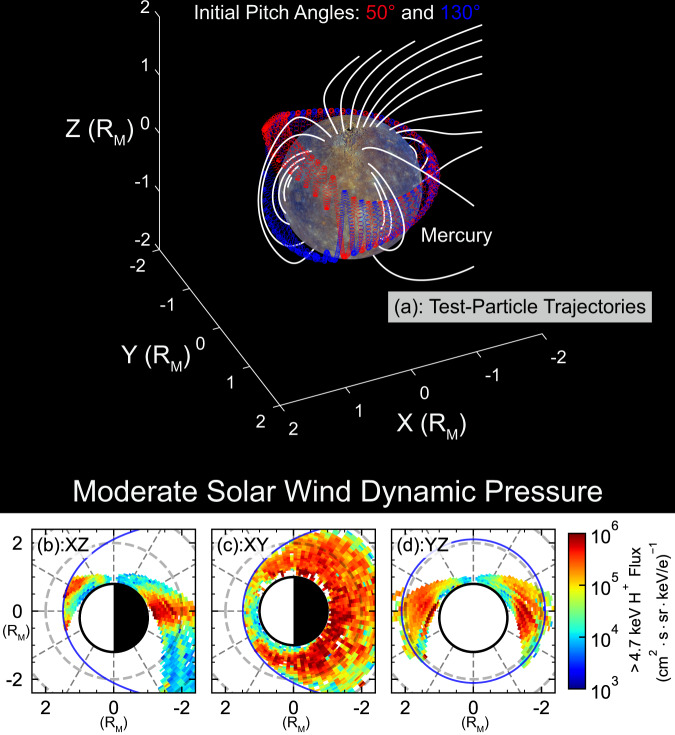
Comparison of the test-particle simulation and MESSENGER observations of Mercury’s off-equatorial ring current. **a** 3D view of the trajectories of the 5 keV test protons shown by the red and blue curves with magnetic field lines shown by the white curves. The model parameters *r*_*Hel*_ and DI are 0.387 AU and 50, respectively, corresponding to a ${{{{{{\rm{R}}}}}}}_{{{{{{\rm{SS}}}}}}}$ of 1.41 ${{{{{{\rm{R}}}}}}}_{{{{{{\rm{M}}}}}}}$. **b**–**d** Energetic proton flux distributions based on MESSENGER observations in the day-night (Local Time: 11–13 h & 23–01 h), geomagnetic equatorial (|*Z*|<0.2 ${{{{{{\rm{R}}}}}}}_{{{{{{\rm{M}}}}}}}$), and dawn-dusk planes (Local Time: 5–7 h & 17–19 h) under moderate solar wind ${p}_{{dyn}}$ ($1.35\ {{{{{{\rm{R}}}}}}}_{{{{{{\rm{M}}}}}}} < {{{{{{\rm{R}}}}}}}_{{{{{{\rm{SS}}}}}}} < 1.49\ {{{{{{\rm{R}}}}}}}_{{{{{{\rm{M}}}}}}}$). The dashed grey circles indicate the gridlines with radius of 2 ${{{{{{\rm{R}}}}}}}_{{{{{{\rm{M}}}}}}}$. See Supplementary Fig. 9 for the colour alternative version of this figure.

A 3D view of the test particle trajectories is shown in Fig. 3a. A closed and bifurcated drift shell is demonstrated in the simulation results. As the test protons drift westward from the magnetotail, the mirror points move poleward, and the bounce path becomes longer. To keep the first adiabatic invariant constant, protons move off the equator to higher latitudes in Mercury’s dayside magnetosphere and form a bifurcated Shabansky orbit^30^. The bifurcation starts at the local time of ~10 h and stops at ~14 h. This local time span can be estimated by the equatorial magnetic field strength distribution: when the equatorial magnetic field strength is even larger than that at the particle mirror point, the particle no longer passes through the equator but moves to a higher latitude of a local magnetic field minimum. The simulation result agrees with the prediction from the magnetic field distribution in the equatorial plane (Supplementary Fig. 2). The bifurcated drift shell spans from $\sim {15}^{\circ }{{{{{\rm{N}}}}}}$ to ${ \sim 45}^{\circ }$N in magnetic latitude and ~1.3 ${{{{{{\rm{R}}}}}}}_{{{{{{\rm{M}}}}}}}$ to ~1.5 ${{{{{{\rm{R}}}}}}}_{{{{{{\rm{M}}}}}}}$ in radial distance within ~10–14 h local time.

Our test-particle simulations suggest the existence of a complete and bifurcated proton ring current under moderate solar wind conditions. This test-particle trajectory is similar to that in previous simulations of 34 keV electrons in Mercury^30^ and 20–300 keV protons in Earth^39^. The simulation results are consistent with observations of the protons trapped in the high latitude regions in Case II. Another simulation under low ${p}_{{dyn}}$ demonstrates an earth-like equatorial ring current, corresponding to the observations in case I, as shown in Supplementary Fig. 3.

### Statistical observational results

Although the test particle simulations strongly indicate a ring current in Mercury’s magnetosphere, further statistical analysis is required to draw and confirm the global picture of the ring current. In this section, we present the superposed spatial distribution of energetic protons with kinetic energy larger than 4.7 keV (4.7–13.3 keV, the top 12 energy channels of FIPS)^43^. These protons are the ring current population with energies close to those in observations and simulations presented in the previous sections. We utilised the superposed analysis method proposed by Zhao et al.^40^ to process FIPS measurements, and the procedures are also described in the Methods, subsection superposed analysis. As indicated in previous simulations, the ring current morphology may depend on the upstream dynamic pressure, and we can apply an additional restriction of the magnetopause subsolar distance to ensure a stable and moderate ${p}_{{dyn}}$. The magnetopause subsolar distance is estimated in the same way (i.e., Shue model) as in the above two cases. Only dayside magnetopause crossings have been used to identify the subsolar distance since they are more sensitive to ${p}_{{dyn}}$ than nightside crossings. The influence of cusp indentation on the estimation of subsolar magnetopause distance is negligible because the high latitude polar crossings of magnetopause are only a very small fraction of all crossings (<2%). Supplementary Fig. 4 presents the histogram of subsolar distances during ~2800 orbits with clear magnetopause crossings and complete particle measurements. These ~2800 orbits are classified into three groups with a similar sample size (low/moderate/high ${p}_{{dyn}}$). In the following analysis, we use observations under moderate ${p}_{{dyn}}$ (i.e., the subsolar standoff distance between 1.35 ${{{{{{\rm{R}}}}}}}_{{{{{{\rm{M}}}}}}}$and 1.49 ${{{{{{\rm{R}}}}}}}_{{{{{{\rm{M}}}}}}}$) to present the proton distribution, and measurements outside the magnetopause are not included.

Figure 3b–d shows the superposed energetic proton fluxes measured by FIPS. As shown, the energetic proton flux in the geomagnetic equatorial plane (Fig. 3c) is higher than 10^5^ ${{{{{{\rm{cm}}}}}}}^{-2}\cdot {{{{{\rm{s}}}}}}{{{{{{\rm{r}}}}}}}^{-1}\cdot {{{{{{\rm{s}}}}}}}^{-1}\cdot ({{{{{\rm{k}}}}}}{{{{{{\rm{eV}}}}}}}/{{{{{\rm{e}}}}}})^{-1}$ in each local time sector except the near noon sector (from 10 h to 14 h local time). In the noon sector, a flux peak appears in northern mid-latitude regions (Fig. 3b), demonstrating an off-equatorial bifurcated ring current, as suggested from the test-particle simulation, with a peak at a radial distance of ~1.4 ${{{{{{\rm{R}}}}}}}_{{{{{{\rm{M}}}}}}}$ and latitude of ~${30}^{\circ }{{{{{\rm{N}}}}}}$. A direct comparison between the observed energetic proton flux and test-particle trajectory in different local time sectors of the meridian plane is presented in the Supplementary Fig. 5. To visualise the difference between bifurcated ring current protons and cusp trapping protons, the trajectory of the modelled cusp trapping proton is overplotted in Supplementary Fig. 5. The trajectory of cusp trapping proton is shown as isolated cyan shaded areas at higher latitudes, while the trajectory of the modelled ring current particle is located closer to the observed flux peaks. The off-equatorial ring current is located near the modelled magnetopause (transparent blue solid line, ${R}_{{SS}}=1.49\ {{{{{{\rm{R}}}}}}}_{{{{{{\rm{M}}}}}}}$) and equatorward to the polar cusp in previous observations^44^. Figure 3d and Supplementary Fig. 5 show that the ring current protons have a higher flux level in the dusk sector than in the dawn sector, which is consistent with previous modelling works (e.g., Exner, Simon^28^, Paral, Trávníček^35^). The spatial distribution of energetic proton fluxes under low and high ${p}_{{dyn}}$ are presented in Supplementary Fig. 6. The corresponding simulation results under low ${p}_{{dyn}}$ are presented in Supplementary Fig. 3 for comparison, which also demonstrates a closed trajectory. As expected, it shows large energetic proton flux around the equator, indicating the appearance of equatorial-crossing energetic protons.

The statistical observational results suggest that energetic protons can be stably trapped and form a complete ring current around Mercury. The energetic protons drift across the noon sector magnetosphere via Shabansky orbit at high latitudes during higher solar wind dynamic pressures or drift across the equatorial plane under weak solar wind forcing near Mercury. Such statistical properties are consistent with our case studies and simulation results shown in Figs. 1, 2, 3 and Supplementary Fig. 3.

**Fig. 4:**
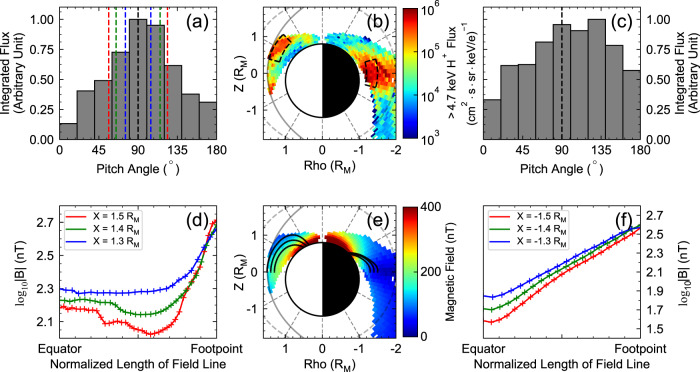
Pitch angle distributions and field line tracing results. **a** The pitch angle distribution of energetic protons in the dayside high latitude magnetosphere (observations inside the area marked by the black box on the dayside in Panel **b**). **b** The spatial distribution of the energetic proton flux in the day-night meridian plane (${{{{{\rm{Rho}}}}}}=X\cdot \sqrt{1+{Y}^{2}/{X}^{2}}$), adopted from Fig. 2b. **c** The pitch angle distribution of energetic protons in the nightside equatorial magnetosphere (observations inside the area marked by the black box on the nightside in Panel **b**. **d** Magnetic field strength variations along the field lines originating from ${{{\it{X}}}}=1.3,1.4,1.5\ {{{{{{\rm{R}}}}}}}_{{{{{{\rm{M}}}}}}}({{{\it{Y}}}}={{{\it{Z}}}}=0)$. **e** Observed mean magnetic field strength distribution (colour plots) and magnetic field line tracing results (the overplotted black lines). **f** Magnetic field strength variations along the field lines originating from ${{{\it{X}}}}=-1.3,-1.4,-1.5\ {{{{{{\rm{R}}}}}}}_{{{{{{\rm{M}}}}}}}({{{\it{Y}}}}={{{\it{Z}}}}=0)$. See Supplementary Fig. 10 for the colour alternative version of this figure.

In addition to the spatial distributions of energetic proton flux, the ${90}^{\circ }$-dominant PAD is also validated by our statistical results in Fig. 4a, c, providing further evidence for stably trapped energetic protons. Figure 4b is adopted from Fig. 3b. Two boxes marked with black dashed lines indicate the areas of interest. Both PADs display a peak at a pitch angle of approximately ${90}^{\circ }$, indicating that the majority of protons are trapped in the dayside off-equatorial region and the nightside equator (Fig. 4a, c). The nightside PAD is much flatter than that on the dayside, which may be due to the effect of current sheet scattering in the nightside plasma sheet^20,40,45^.

Besides, the local magnetic field minimum is an essential condition for off-equatorial trapping. The spatial distribution of the observed magnetic field strength (Fig. 4e) reveals a magnetic field off-equatorial minimum at ~${30}^{\circ }{{{{{\rm{N}}}}}}$ on the dayside. By tracing the observed magnetic field lines (blue, green and red solid lines) originating from ${{{{{\rm{X}}}}}}=1.3,1.4,1.5{{{{{{\rm{R}}}}}}}_{{{{{{\rm{M}}}}}}}$(Y = Z = 0, see Methods, subsection field line tracing), the magnetic field strength along the field lines is obtained (Fig. 4d). The equatorward loss cones can be determined by $\left({{{{{\rm{Eq.}}}}}}\; (2):\alpha ={{\arcsin }}\left(\sqrt{{B}_{{\min }}/{B}_{{equator}}}\right)\right)$^46^, which are presented with red, green, and blue dashed lines in Fig. 4a. As expected, the energetic proton flux out of the loss cone is higher than from within the loss cone, indicating that the majority of the observed protons are trapped in Northern Hemisphere (Fig. 4a). Similar field line tracing results and PADs in the nightside magnetosphere are presented in Fig. 4c, e, f, revealing an ~${90}^{\circ }$-dominant PAD and equatorial magnetic minima on the nightside.

**Fig. 5:**
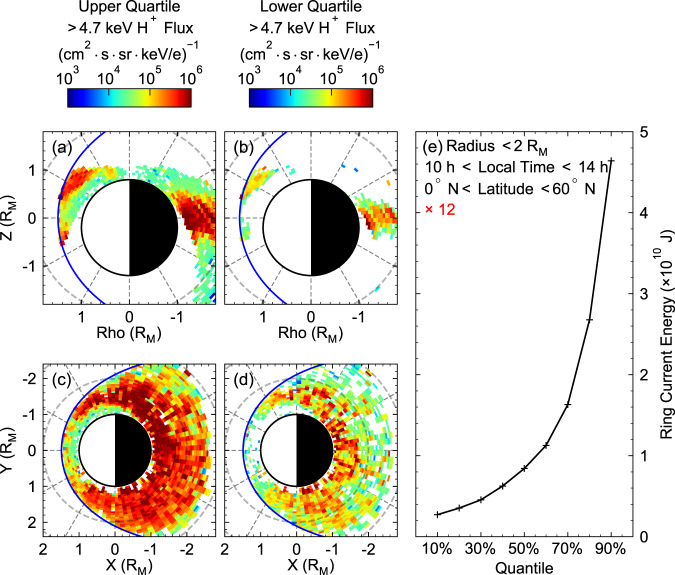
Ring current variability and its total energy content under moderate solar wind conditions. **a**, **b** Distribution of the upper and lower quartiles of the energetic proton flux in the day-night meridian plane (${{{{{\rm{Rho}}}}}}=X\cdot \sqrt{1+{Y}^{2}/{X}^{2}}$). **c**, **d** Distribution of the upper and lower quartiles of the energetic proton flux in the equatorial plane. **e** Quantile distribution of the ring current proton’s total energy. See Supplementary Fig. 11 for the colour alternative version of this figure.

After the confirmation of the existence of Mercury’s ring current, we now intend to determine the dynamic variability of the Mercury’s ring current and its total energy content. Fig. 5a–d shows the distributions of the upper and lower quartiles of the energetic proton flux in the day-night meridian and equatorial planes under moderate solar wind conditions. The ring current’s dynamic variability can be clearly shown by the remarkable difference between the quartiles. We then estimate the quantile distribution of the total energy content within the ring current by integrating the energy density inside the ring current region (see Methods, subsection estimation of ring current energy). The integration domain is restricted in both magnetic latitude (<${60}^{\circ }$) and local time (10–14 h) to exclude the energy contribution from the polar cusp and nightside plasma sheet, respectively. Moreover, this integration only considers the measurements from Northern Hemisphere due to the poor data coverage in Southern Hemisphere. Thus, we multiply the integrated energy by 12 to include the contributions from all the local time sectors and both hemispheres. The final calculated total energy content ranges from ~2 × ${10}^{9}$ J to ~5 × ${10}^{10}$ J (10–90% quantile), as shown in Fig. 5e. The large difference between the 10% and 90% quantiles of the ring current proton energy, as shown in Fig. 5e, indicates the enormous dynamic variability of Mercury’s ring current^47^. We look forwards to a deeper understanding of Mercury’s ring current energy and its variability from the upcoming BepiColombo observations with superior orbital coverage in both hemispheres and the comprehensive energy range of charged particle measurement^48^.

## Discussion

Our statistical results present comprehensive observational evidence of the existence of ring currents in Mercury’s magnetosphere. The ring current exists not only under nominal solar wind dynamic pressure, as suggested by Exner, Simon^28^, but also under more intense solar wind conditions, which does not appear in the previous hybrid and MHD model results^26,27^. And unlike Earth’s dayside ring current located around the magnetic equator, Mercury’s ring current is bifurcated in the dayside meridian plane due to the strong poleward magnetic mirror force near the magnetic equator under moderate dynamic pressure. Test particle simulations also reproduce a bifurcated proton trajectory, which precisely coincides with the observed distributions. Such a bifurcation of ring current particle trajectory was first suggested by Shabansky in Earth’s dayside outer magnetosphere^29^. The magnetopause current largely enhances the dayside magnetic field inside the magnetosphere. As a result, a local maximum in the magnetic field strength is produced at the subsolar equator, leading to a poleward magnetic mirror force that prevents charged particles from crossing the equator. Therefore, these particles can be locally trapped near the off-equatorial minimum in one hemisphere and constitute a bifurcated particle distribution.

The bifurcation of the drift shell only occurs near the dayside magnetopause (*L* > 7) in the terrestrial magnetosphere and does not significantly affect the ring current and the radiation belt^39^. However, due to the stronger compression and weaker dipole magnetic field in Mercury’s magnetosphere, this bifurcation is a fundamental feature of Mercury’s ring current under moderate and strong solar wind conditions. Both the simulations (Fig. 3a) and the observed statistical distributions (Fig. 3b, c, d) suggest that the bifurcation spans from ~10 h to ~14 h local time and energetic protons are trapped near the off-equatorial minima at ~${30}^{\circ }.$ In other local time sectors, the protons exhibit an Earth-like drift-bounce signature with a longer bounce path on the dayside than on the nightside. The bifurcation tends to vanish when ${p}_{{dyn}}$ decreases^22^. In this situation, some ring current protons have equatorial drift orbits similar to those on Earth. Overall, Mercury’s ring current is bifurcated under most solar wind conditions. As Mercury’s orbit has an eccentricity of ~0.20, the seasonal variation in ${p}_{{dyn}}$ and Mercury’s ring current is expected to be another significant signature.

Mercury’s miniature magnetosphere enables a large fraction of energetic protons to be lost through magnetopause shadowing and surface absorption during their drifting. Consequently, energetic protons are mainly distributed on the dusk side, similar to Earth’s partial ring current. This dawn-dusk asymmetry is also visible in our observation of Mercury’s ring current (Fig. 3d), indicating the possible loss of ring current protons.

In addition to the nightside plasma sheet proton, cusp trapped proton is another probable source of the ring current during weak solar wind enforcement^34^. Opposite transport (i.e., from the ring current to the cusp trapping region) may occur after a sudden enhancement in solar wind ${p}_{{dyn}}$^49^. Additionally, planetary ions such as sodium ions may also be a significant carrier of the ring current, as indicated by simulation results in Exner et al.^28^, Paral et al.^35^, and Yagi et al.^36^. However, based on the in situ measurements from FIPS onboard MESSENGER spacecraft^44,50^, the contribution of sodium ions to the total kinetic energy is much smaller than that of protons. The relative contributions of different planetary ion species to the ring current require further study, and the investigations based on BepiColombo observations will further help in understanding the underlying effects^48^.

The total energy carried by the magnetospheric ring current is estimated to be ~${0.2-5\times 10}^{10}$ J by integrating the observed proton thermal pressure within 2 ${{{{{{\rm{R}}}}}}}_{{{{{{\rm{M}}}}}}}$ in the dayside sector. Alternatively, the total current inside the ring current can be roughly estimated to be ~$1\ {{{{{\rm{kA}}}}}}-31\ {{{{{\rm{kA}}}}}}\;\left({{{{{\rm{Eq.}}}}}}\; (3):I_{L}=\frac{3{U}_{L}L}{2\pi {B}_{M}{R}_{M}^{2}}{|}_{L=1.5}\right)$^46^ in a simple dipole treatment, which is comparable to the field-aligned current at Mercury^28,51–53^. According to the DPS relation^54,55^, this proton ring current can cause a magnetic depression of ~$0.2\ {{{\rm{nT}}}} -3.5$ nT at the ring current’s centre, which is significant compared to the magnetic field strength of Mercury. The ~0.2 nT$-$3.5 nT decrease ($\sim 0.4 \% -2.5 \%$) in the magnetic field strength is equivalent to a geomagnetic storm with Dst ranges from $-32\ {{{\rm{nT}}}}\; {{{\rm{to}}}}-556$ nT on Earth. Because of this relatively intense ring current, Mercury may also have magnetic storms in some sense. However, unlike the Dst index on Earth, the observable magnetic depression at the surface of Mercury may vary from 30% (south pole) to 250% (magnetic equator) of the DPS estimation because Mercury has a comparable radius to the size of the ring current. Thus, the magnetic depression may be even larger at the equatorial plane of Mercury. However, it is difficult to measure this magnetic depression because the magnetopause current enhances the surface magnetic field, which is the opposite of the ring current contribution, and the contribution from other sources, including the field-aligned current and induction effect, could be even larger than that from the ring current during active times^27,28,36,56,57^.

To summarise, in this study, we demonstrate the existence of Mercury’s ring current by providing both observational and simulation evidence. The ring current has a bifurcated morphology caused by the proton’s Shabansky orbit. The estimated total energy carried by the ring current is ~${0.2-5\times 10}^{10}$ J, which would trigger a magnetic storm with a magnetic field depression of $\sim 0.2-3.5{{{{{\rm{nT}}}}}}$. We also expect a series of drift-related phenomena in observations from the JAXA-ESA BepiColombo mission when it begins orbiting Mercury in 2025^48^.

## Methods

### Instrumentation

Observational data used in this study are measured by the Fast Image Plasma Spectrometer (FIPS) and the Magnetometer (MAG) onboard MESSENGER.

MAG is a fluxgate magnetometer that measures the magnetic field vectors with a frequency of 20 Hz. FIPS consists of an electrostatic analyser and a time-of-flight sensor. It measures the differential flux of ions with energy between 46 eV/e and 13.3 keV/e at a time resolution of ~10s^43^. MAG and FIPS measurements can produce energy-resolved pitch angle distributions per minute. However, FIPS only has an effective field of view of ~1.15${{{{{\rm{\pi }}}}}}$. The parallel and anti-parallel pitch angle channels are always blocked, as shown by the grey boxes in Figs. 1b and 2b.

### Aberrated MSM coordinates

In this study, we use the aberrated Mercury-Sun magnetospheric (aMSM) coordinate system. In traditional MSM coordinates, the X-axis and Z-axis point to the sun and north pole, respectively, and the Y-axis completes a right-hand system. In the aberrated coordinates, Mercury’s orbital velocity is considered. The X-axis is anti-parallel to the solar wind direction in the rest of the reference frame of Mercury. The aberration angle varies between $-{5.5}^{\circ }$ and $-{8.4}^{\circ }$ assuming a solar wind speed of 400 km/s.

### Limitations of KT17 model

1. The KT17 model is a static model that does not take the temporal variation of the magnetic field into consideration.
2. The KT17 model only has two input parameters, the heliocentric distance of Mercury in astronomical unit and the disturbance index. The disturbance index, which is derived from the magnetic fluctuation intensity, may reflect some IMF information due to the relationship between magnetospheric fluctuation and IMF. However, this model is not explicitly controlled by the interplanetary magnetic field (IMF).
3. The accuracy of the KT17 model outside MESSENGER’s spatial coverage is still questionable due to the lack of comparison between the model and observations.
4. The electric current system in the KT17 model consists of the magnetopause and magnetotail current. While the contributions from induction effects^56,57^, region-I field-aligned current^52^, etc. are not included.

### Superposed analysis

Since the dipole field and the ring current have axial symmetry to some extent, a 2-dimensional polar coordinate grid is used. The resolution of the radial distance and polar angle is 0.05 ${{{{{{\rm{R}}}}}}}_{{{{{{\rm{M}}}}}}}$ and ${3.75}^{\circ }$, respectively. In the analysis of the distribution at the equatorial plane, the third axis (i.e., the Z-axis) is limited between −0.2 ${{{{{{\rm{R}}}}}}}_{{{{{{\rm{M}}}}}}}$ and 0.2 ${{{{{{\rm{R}}}}}}}_{{{{{{\rm{M}}}}}}}$. This range is close to the thickness of Mercury’s cross-tail current sheet. Grid boxes in the day-night (dawn-dusk) meridian plane are limited to [11 h, 13 h] and [23 h, 01 h] ([5 h, 7 h] and [17 h, 19 h]) in local time to ensure that there are enough samples in all grid boxes. By accumulating FIPS and MAG measurements in each grid box, a superposed, averaged measurement of the proton flux and magnetic field strength is obtained.

### Field line tracing

We include a 2-D field line tracing method in this section to trace magnetic field lines in the day-night meridian plane. The third dimension, local time, is limited by the ranges of [11 h, 13 h] and [23 h, 01 h] for the dayside and nightside, respectively. Here, the solar wind parameters except ${p}_{{dyn}}$ are not classified, meaning that the traced magnetic field direction and intensity are a superposition of the observed magnetic field under different upstream conditions. Thus, our trace results represent the mean magnetic field configuration under certain solar wind dynamic pressures.

Step 1: A position is given in polar coordinates ${{{{{{\bf{r}}}}}}}_{{{{{{\boldsymbol{0}}}}}}}=({\rho }_{0},{z}_{0})$.

Step 2: Calculate the average magnetic field within $0.1{{{{{{\rm{R}}}}}}}_{{{{{{\rm{M}}}}}}}$ around ${{{{{{\bf{r}}}}}}}_{i}.$

$${{{{{{\bf{B}}}}}}}_{i}=\left({B}_{{{{{{\rm{\rho }}}}}},i},{B}_{{{{{{\rm{z}}}}}},i}\right)= < {{{{{{\bf{B}}}}}}}_{j} > =( < {B}_{{{{{{\rm{\rho }}}}}},j} > ,{ < B}_{{{{{{\rm{z}}}}}},j} > )$$

where $|{{{{{{\bf{r}}}}}}}_{j}-{{{{{{\bf{r}}}}}}}_{i}|=\sqrt{{\left({\rho }_{j}-{\rho }_{i}\right)}^{2}+{\left({z}_{j}-{z}_{i}\right)}^{2}} \; < \;0.1\ {{{{{{\rm{R}}}}}}}_{{{{{{\rm{M}}}}}}}$, and the angle bracket (< >) indicates the average.

Step 3: $${{{{{{\bf{r}}}}}}}_{i+1}={{{{{{\bf{r}}}}}}}_{i}+\delta l\cdot \frac{{\vec{{{{{{\bf{B}}}}}}}}_{i}}{\left|{{{{{{\bf{B}}}}}}}_{i}\right|}$$, where $${{{{{\rm{\delta }}}}}}l=0.05\ {{{{{{\rm{R}}}}}}}_{{{{{{\rm{M}}}}}}}$$.

Step 4: If ${{{{{{\bf{r}}}}}}}_{i+1}$ is below the planetary surface of Mercury, break; otherwise, return to Step 2.

### Estimation of ring current energy

To estimate the total energy carried by the ring current, we numerically integrated the thermal pressure inside the grid boxes with local time between 10 h and 14 h and magnetic latitude between ${0}^{\circ }{{{{{\rm{N}}}}}}$ and ${60}^{\circ }{{{{{\rm{N}}}}}}$ using the following two equations.

$${{{{{{\rm{W}}}}}}}_{{{{{{{\rm{H}}}}}}}^{+}}=2\times 6\times \int\nolimits_{-30^{\circ }}^{30^{\circ}}\int\nolimits_{90^{\circ}}^{30^{\circ}}\int \nolimits_{1{{{{{{\rm{R}}}}}}}_{{{{{{\rm{M}}}}}}}}^{2{{{{{{\rm{R}}}}}}}_{{{{{{\rm{M}}}}}}}}{p}_{{{{{{\rm{th}}}}}}}\left(r,\theta ,\phi \right)\cdot {r}^{2}{{\sin }}\;\lambda\; {drd}\lambda d\phi$$

$${{{{{{\rm{W}}}}}}}_{{{{{{{\rm{H}}}}}}}^{+}}=12\times {\Sigma }_{i,j,k}\;{p}_{{{{{{\rm{th}}}}}}}({r}_{i},{\theta }_{j},{\phi }_{k})\cdot {r}_{i}^{2}\;{{\sin }}\;{\lambda }_{j}\Delta r\Delta \lambda \Delta \phi$$

where ${p}_{{{{{{\rm{th}}}}}}}$ is the thermal pressure of protons detected by FIPS, ${r}_{i},{\lambda }_{j},{\phi }_{k}$ are the radial distance, latitude, and azimuthal angle (i.e.,$.\phi ={{\arctan }}\left(\frac{{Y}_{{aMSM}}}{{X}_{{aMSM}}}\right)$, $\phi =-{30}^{\circ }$ and $\phi ={30}^{\circ }$ correspond to local times of 14 h and 10 h, respectively) of the grid boxes with indices of [*i, j, k*].

By choosing different quantiles of the thermal pressure inside each grid box, the quantile distribution of the ring current is presented in Fig. 5e.

### Test particle simulations

To investigate the ring current morphology, we performed test-particle simulations with KT17 magnetic field model. The test particle simulations in this work are implemented by the following 6 main steps.

Step 1: Input initial kinetic, pitch angle, and position$({{{{{{\bf{r}}}}}}}_{0})$ of test particle.

Step 2: Input model parameters, heliocentric distance (${r}_{{hel}}$) and disturbance index (DI).

Step 3: Calculate the initial velocity in the aMSM coordinates.

Step 4: Calculate the model magnetic field ${{{{{{\bf{B}}}}}}}_{{{{{{\bf{i}}}}}}}$ and local gyro frequency, ${\tau }_{i}$ according to the particle position ${{{{{{\bf{r}}}}}}}_{{{{{{\rm{i}}}}}}}$.

Step 5: Update particle’s velocity and position (${{{{{\bf{v}}}}}}$_i_ and ${{{{{\bf{r}}}}}}$_i_) according to the motion equations with 4^th^ order Runge–Kutta method and time step of 1/1000 ${\tau }_{i}$.

Step 6: If ${{{{{{\bf{r}}}}}}}_{{{{{{\rm{i}}}}}}+1}$ is below the planetary surface of Mercury or out of the magnetopause, break; otherwise return to Step 4.

The simulation results are presented in Fig. 3a. The Fortran implementation of the above algorithm is provided with this paper^58^.

## Supplementary information

Supplementary Information

## Data Availability

MESSENGER data used in this study are available from the Planetary Data System (PDS): https://pds.nasa.gov; Magnetometer: https://pds-ppi.igpp.ucla.edu/search/view/?f=yes&id=pds://PPI/MESS-E_V_H_SW-MAG-3-CDR-CALIBRATED-V1.0 and Fast Imaging Plasma Spectrometer: https://pds-ppi.igpp.ucla.edu/search/view/?f=yes&id=pds://PPI/MESS-E_V_H_SW-EPPS-3-FIPS-DDR-V2.0. Source data are provided with this paper. The datasets generated during and/or analysed during the current study are available from the corresponding author on reasonable request. Source data are provided with this paper.

## Source data

Source Data

## Code availability

The test particle simulation code (in Fortran) is available at GitHub^58^ (https://github.com/jt-zhao/Mercury-s-Ring-Current).
