# Supplementary material for: Observational evidence of ring current in the magnetosphere of Mercury
Source: Nat Commun. 2022 Feb 17;13:924. doi: 10.1038/s41467-022-28521-3 (PMC8854437; doi:10.1038/s41467-022-28521-3)
Supplement: Supplementary file 1 — Supplementary Information [file 41467_2022_28521_MOESM1_ESM.pdf]

Supplementary Information for

Observational evidence of ring current in the magnetosphere of Mercury

J.-T. Zhao[1], Q.-G. Zong[1, 2*], C. Yue[1*], W.-J. Sun[3], H. Zhang[4], X.-Z. Zhou[1], G. Le[5], R. Rankin[6], J. A. Slavin[3], J. M. Raines[3], Y. Liu[1], and Y. Wei[7].

[1]Institute of Space Physics and Applied Technology, Peking University, Beijing, China.

[2]Polar Research Institute of China, Shanghai, China.

[3]Department of Climate and Space Sciences and Engineering, University of Michigan, Ann Arbor, Michigan 48109, USA.

[4]Geophysical Institute, University of Alaska Fairbanks, AK 99775, USA.

[5]ITM Laboratory, Heliophysics Science Division, NASA Goddard Space Flight Center, Greenbelt, Maryland, USA.

[6]Department of Physics, University of Alberta, Edmonton T6G2R3, Canada.

[7]Institute of Geology and Geophysics, Chinese Academy of Sciences.

* These authors jointly supervised this work: Q.-G. Zong; C. Yue

Email: qgzong@pku.edu.cn; yuechao@pku.edu.cn

Supplementary Figs. 1-16

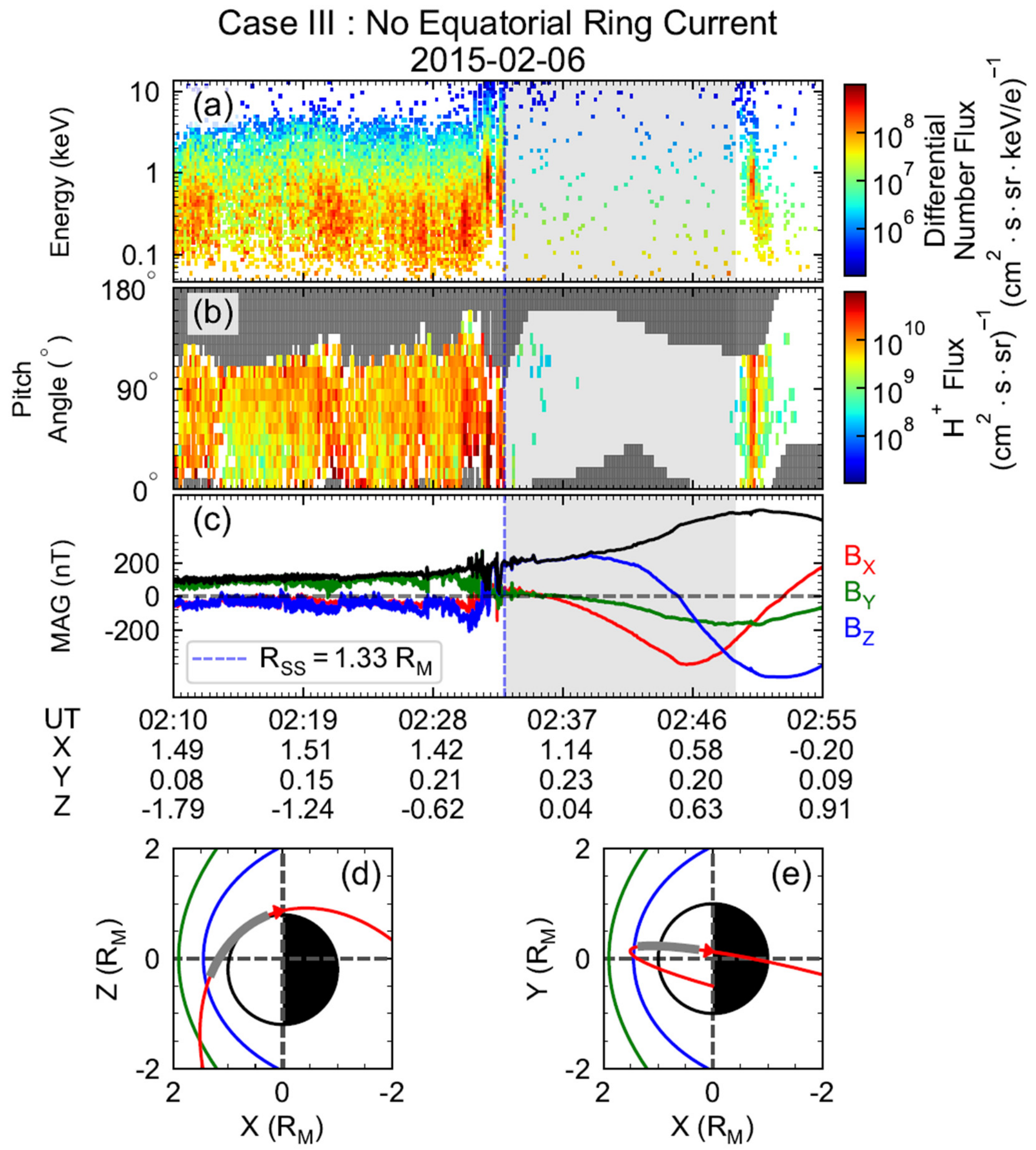

Supplementary Fig. 1. **MESSENGER's observations during dayside magnetosphere crossing without ring current under strong solar wind forcing on Feb 6th, 2015.** (a) Energy spectrum of the proton. (b) Corresponding pitch angle distribution, with uncovered pitch angle bins noted by grey grids. (c) Magnetic field vectors (red, green, and blue solid lines represent the B_x, B_y and B_z components, respectively) and strength (black solid line). (d, e) the trajectory of MESSENGER in XZ, XY planes. The thick shaded area indicates the time period during which MESSENGER passes through the dayside magnetosphere of Mercury without sensible proton flux enhancement. The corresponding spacecraft locations during this interval are plotted in (d, e) as grey bold lines. See Supplementary Fig. 12 for the colour alternative version of this figure.

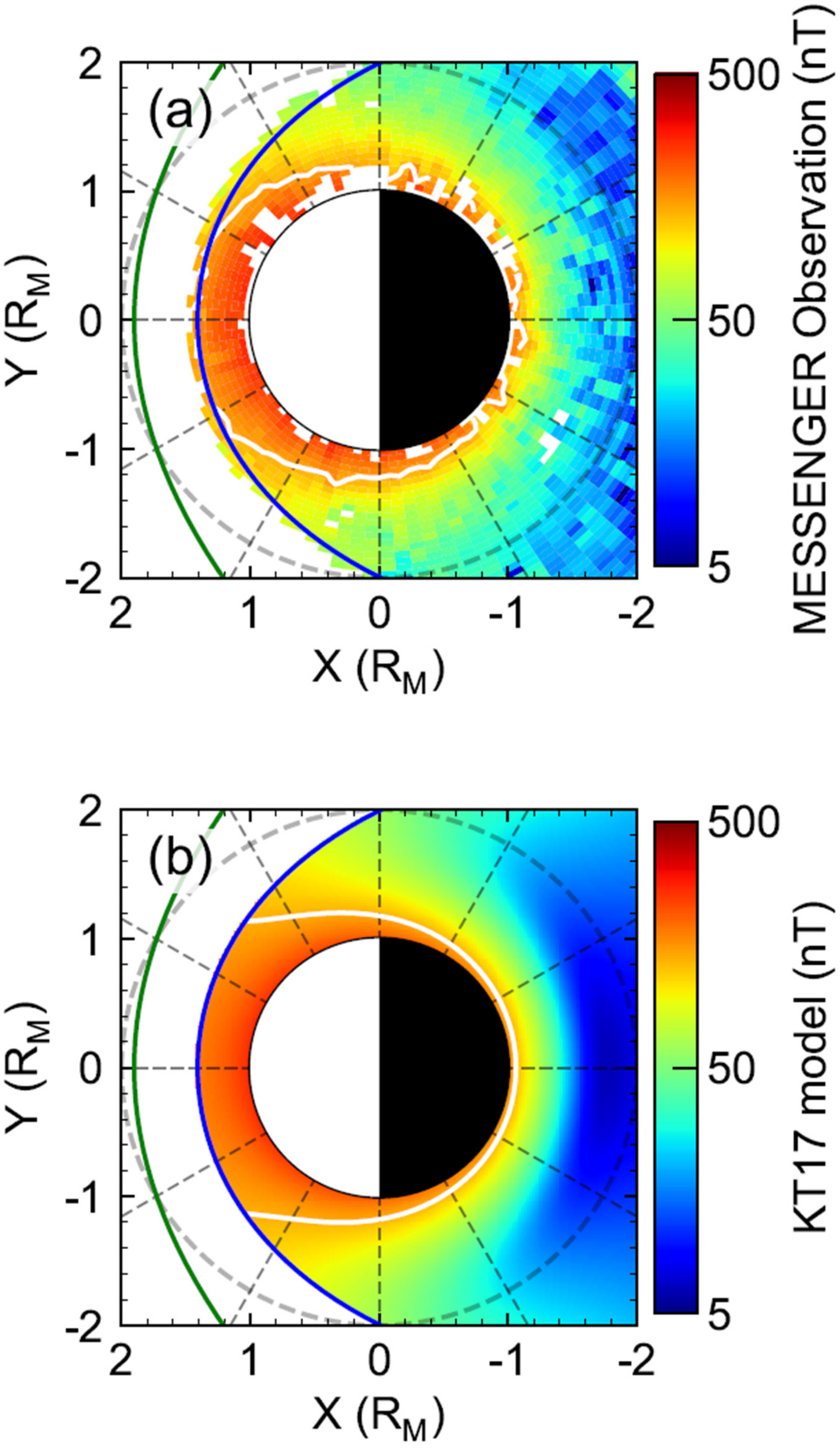

Supplementary Fig. 2. **Magnetic field strength distributions on the equatorial plane.** (a) The observed mean magnetic field around the geomagnetic equator ($|Z| < 0.2\ R_M$). (b) KT17 magnetic field model ($r_{Hel} = 0.387\ \text{AU}, \text{DI} = 50$). Blue and green solid lines represent the modelled magnetopause and bow shock, respectively. The white solid line is the contour of the magnetic field with a strength of 140 nT, close to the magnetic field strength at the mirror point of our initial test particle. See Supplementary Fig. 13 for the colour alternative version of this figure.

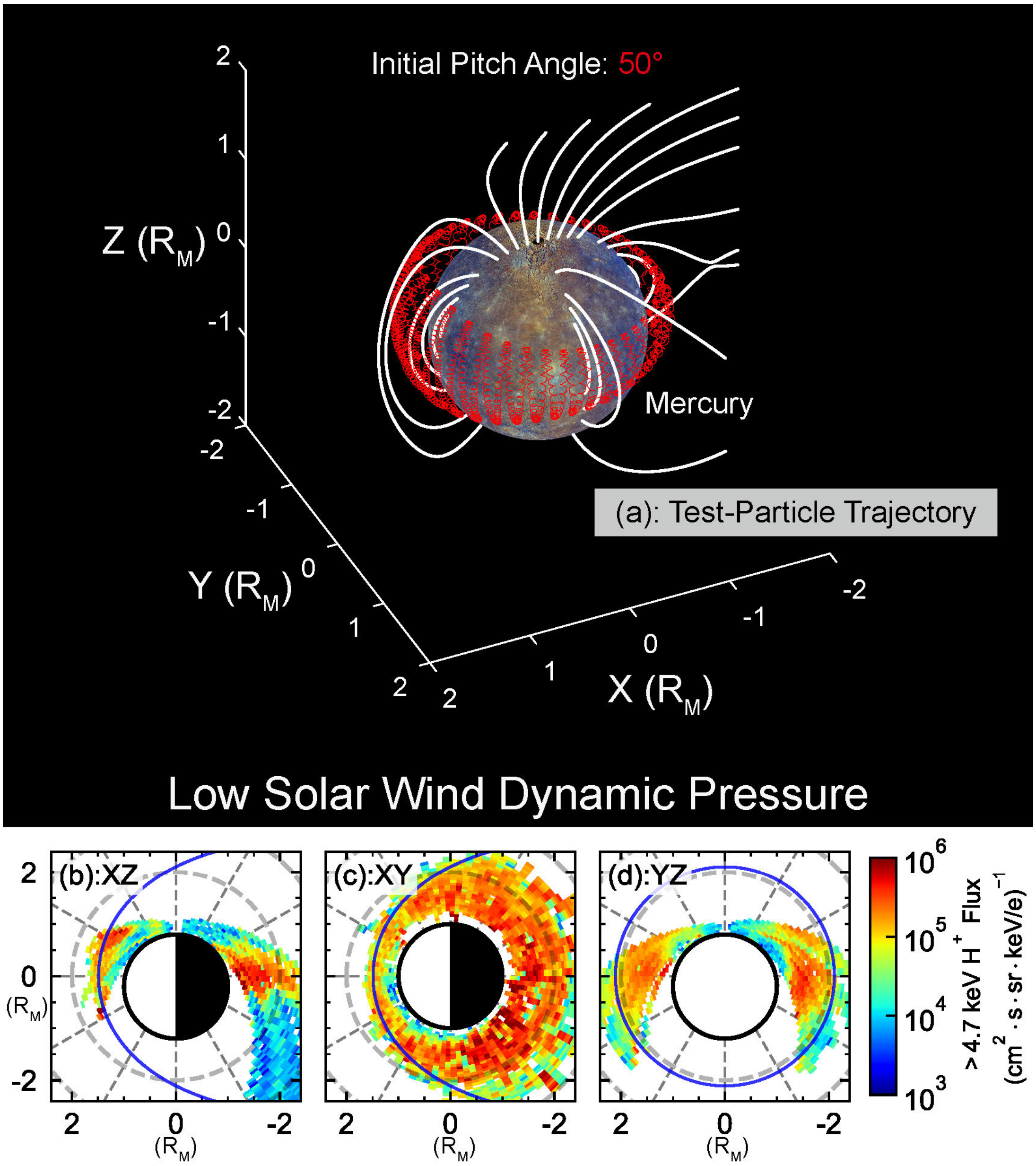

Supplementary Fig. 3. **Comparison of the test-particle simulation and MESSENGER observations of Mercury's equatorial ring current.** (a) 3D view of the trajectory of the test proton shown by the red curve with magnetic field lines shown by the white curves. The model parameters r_{Hel} and DI are 0.467 AU and 0, respectively, corresponding to a R_{SS} of 1.62 $\mathrm{R_M}$. (b, c, d) Energetic proton flux distributions based on MESSENGER observations on the day-night (local time: 11 h-13 h & 23 h -01 h), geomagnetic equatorial ($|Z| < 0.2$ $\mathrm{R_M}$), and dawn-dusk planes (local time: 5 h-7 h & 17 h-19 h) under low solar wind p_{dyn} ($1.49\mathrm{R_M} < R_{SS}$). See Supplementary Fig. 14 for the colour alternative version of this figure.

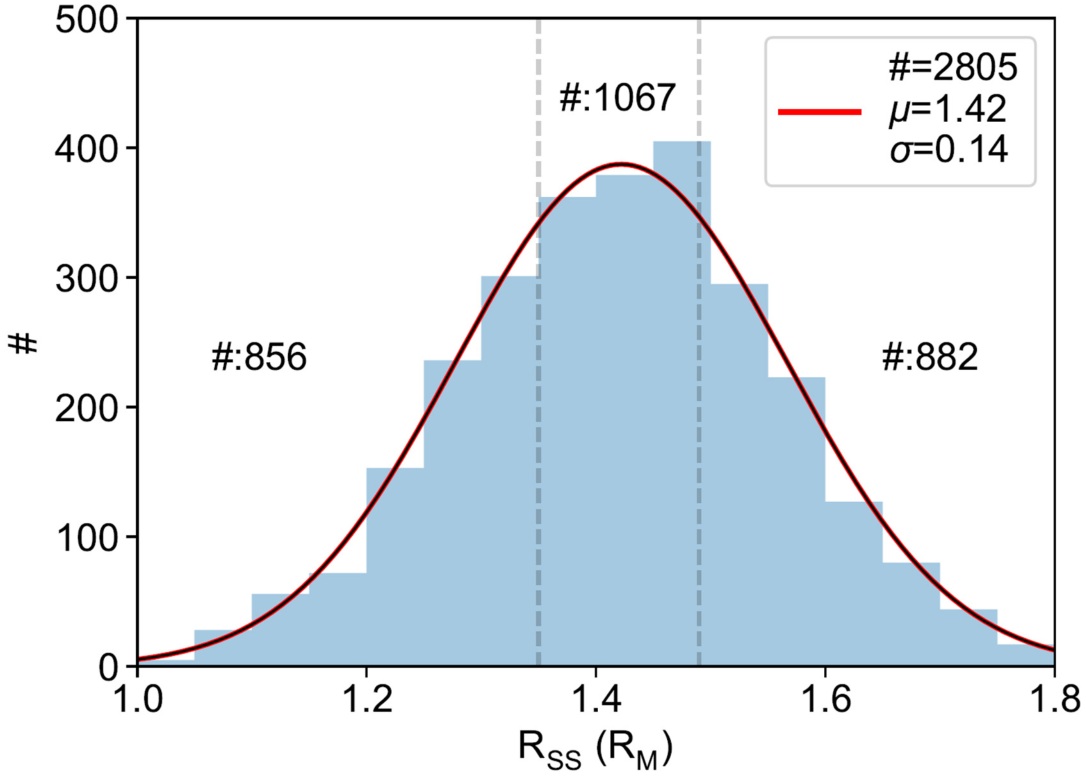

Supplementary Fig. 4. **The histogram of subsolar distances with a total of 2805.** The mean and standard derivation are 1.42 $\mathrm{R_M}$ and 0.14 $\mathrm{R_M}$, respectively. A Gaussian distribution with the same parameters is denoted as the red solid line. The grey dashed lines indicate the critical values of classification (i.e. $R_{SS} = \mu - \frac{\sigma}{2}$ and $R_{SS} = \mu + \frac{\sigma}{2}$). The event numbers in each group [low ($R_{SS} < 1.35$)/medium ($1.35 < R_{SS} < 1.49$)/high ($R_{SS} > 1.49$)] are noted above the Gaussian curve.

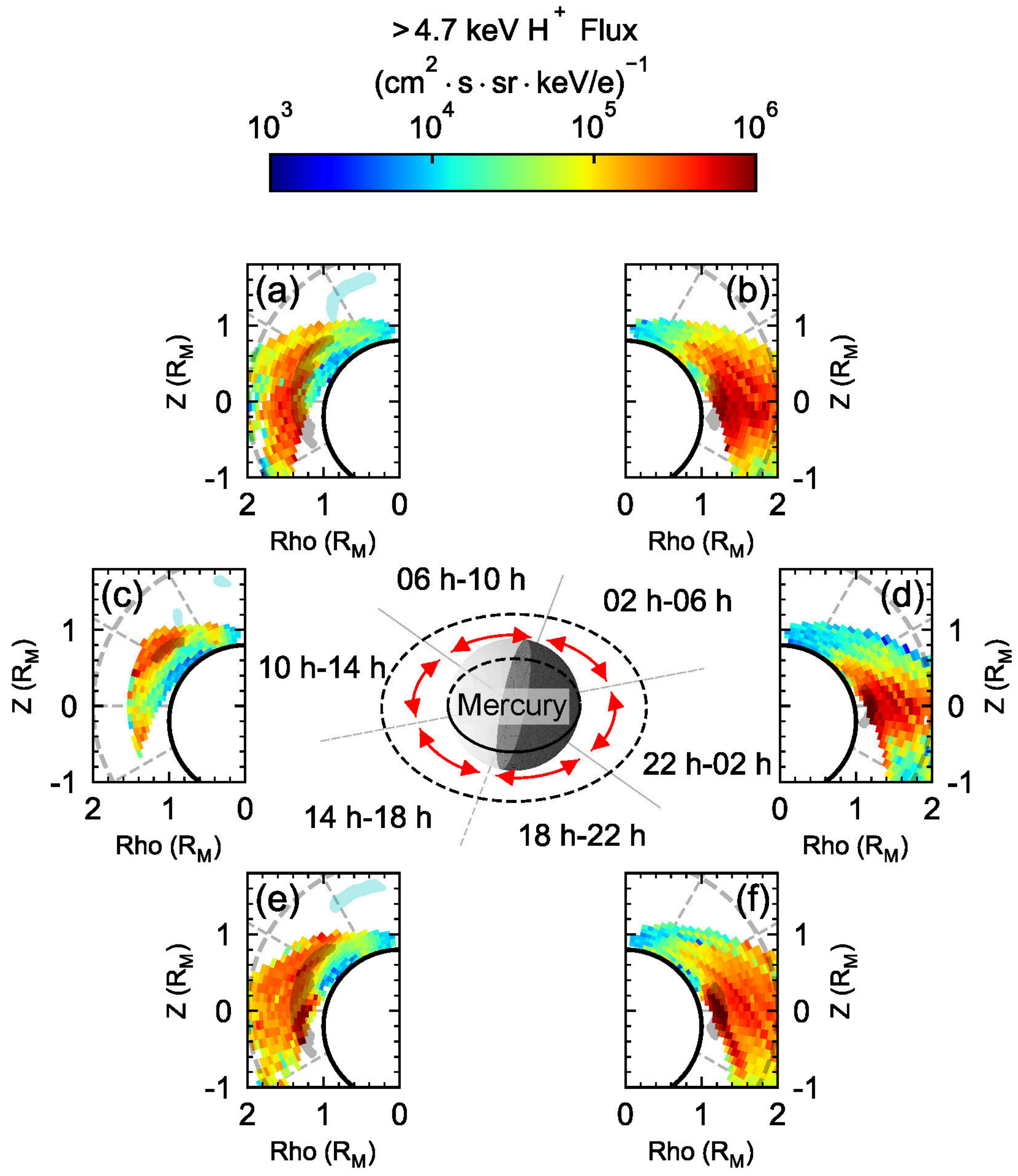

Supplementary Fig. 5. **Comparison of the test particle trajectories and MESSENGER observations.** (a-f) Statistical distributions of energetic proton flux in the meridian plane within different local time ranges. The ring current particle trajectory is overplotted as a grey area. The cusp trapped proton trajectory is overplotted as a cyan area for comparison. See Supplementary Fig. 15 for the colour alternative version of this figure.

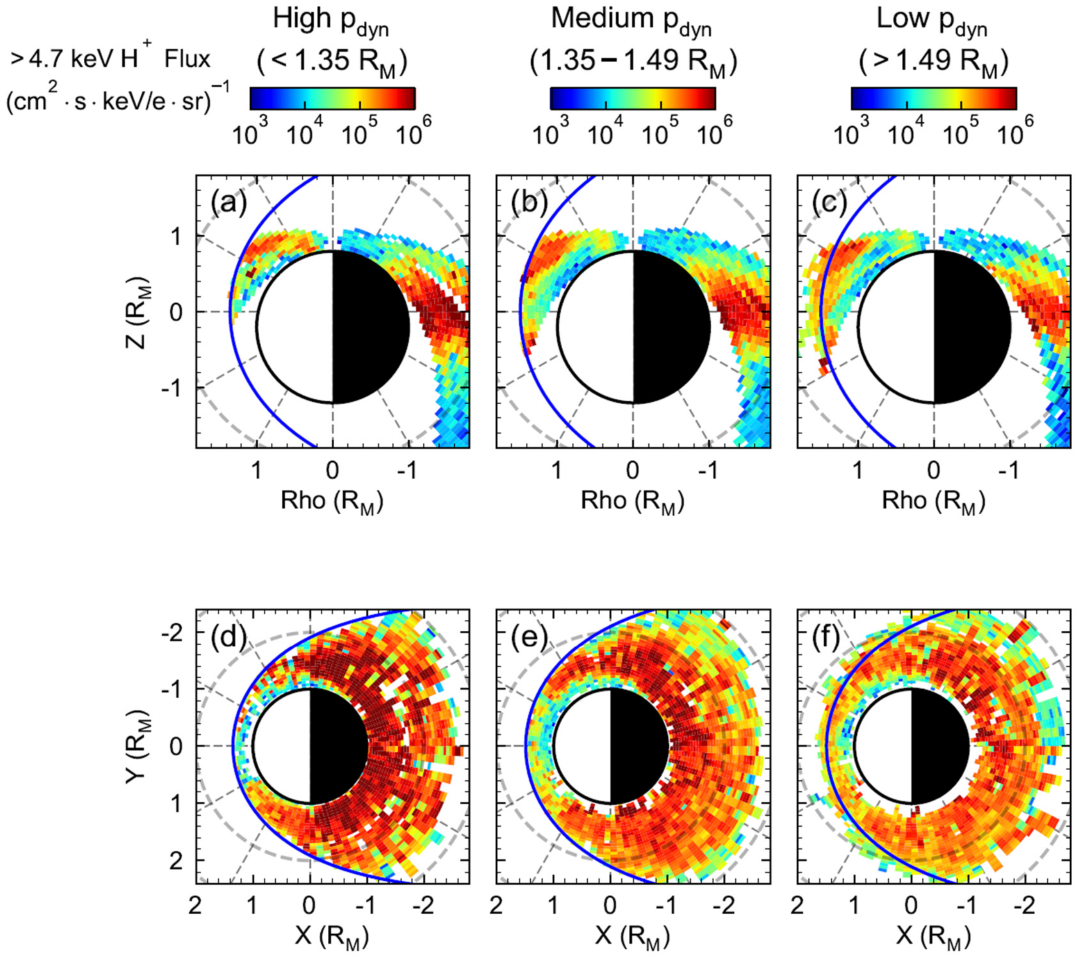

Supplementary Fig. 6. **Distributions of energetic proton fluxes in units of $\mathbf{cm^{-2} \cdot s^{-1} \cdot sr^{-1} \cdot (keV/e)^{-1}}$ under different solar wind forcing conditions.** (a-c) Energetic proton flux distributions in the day-night plane (local time: 11 h-13 h & 23 h -01 h) under high, moderate, and low solar wind forcing conditions. (d-f) Energetic proton flux distributions in the geomagnetic equatorial planes ($|Z|<0.2$ R_M) under high, moderate, and low solar wind forcing conditions. See Supplementary Fig. 16 for the colour alternative version of this figure.

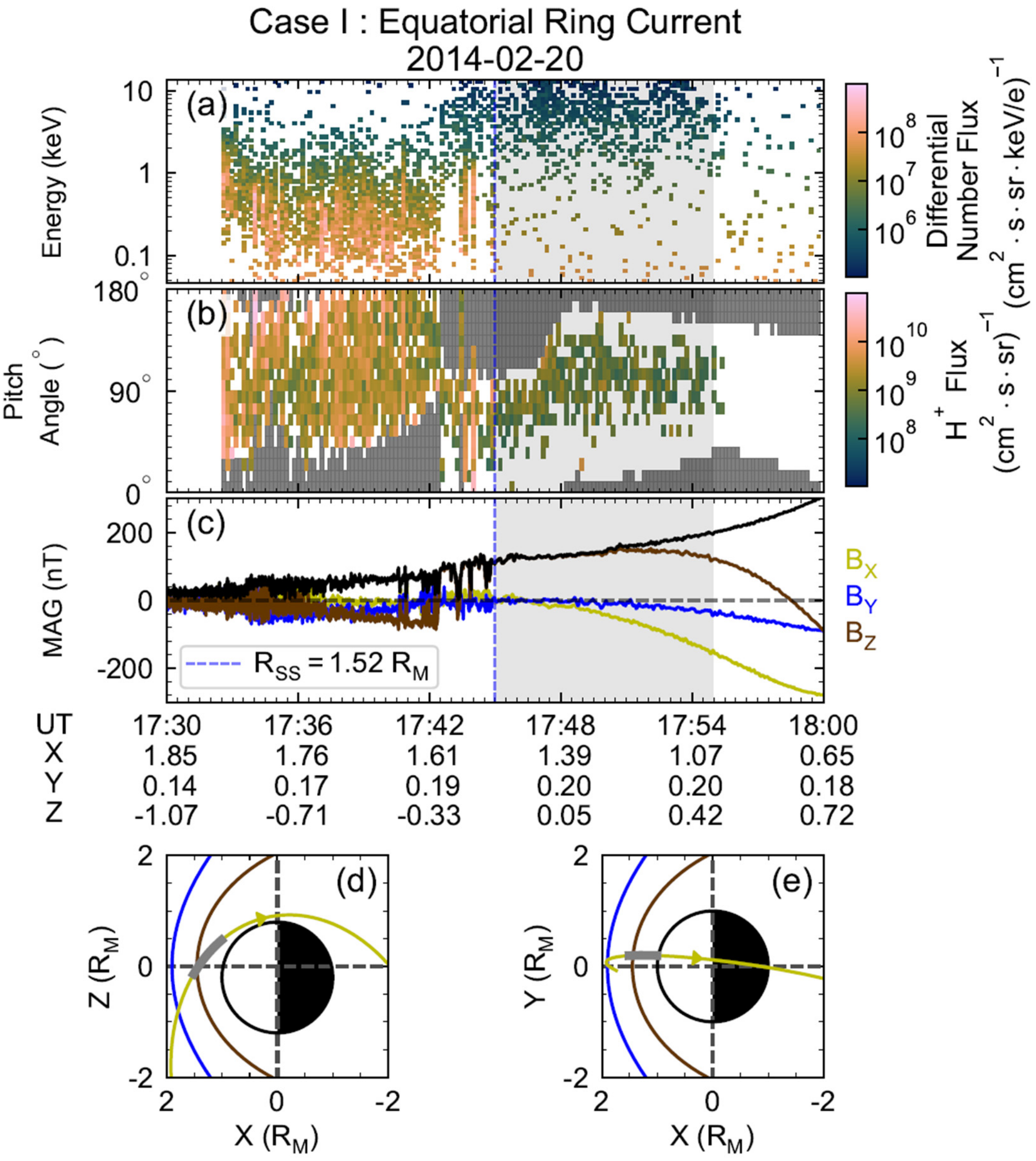

Supplementary Fig. 7. **MESSENGER observations during dayside magnetosphere crossing with potential equatorial ring current on Feb 20th, 2014 (Color alternative[59] version of Figure 1).** (a) Energy spectrum of the protons. (b) Corresponding pitch angle distribution, with uncovered pitch angle bins noted by grey grids. (c) Components of the magnetic field (yellow, blue, and brown solid lines represent the B_x, B_y and B_z components, respectively) and strength (solid black line). (d-e) the trajectory of MESSENGER in XZ, XY planes. The thick shaded areas in (a-c) indicate the time periods during which FIPS observed trapped energetic protons. The corresponding spacecraft locations during these intervals are plotted in (d-e) as bold grey lines, and the blue and brown curves show the modelled dayside bow shock and magnetopause that is obtained from the statistical distribution of observed crossing points[12], respectively.

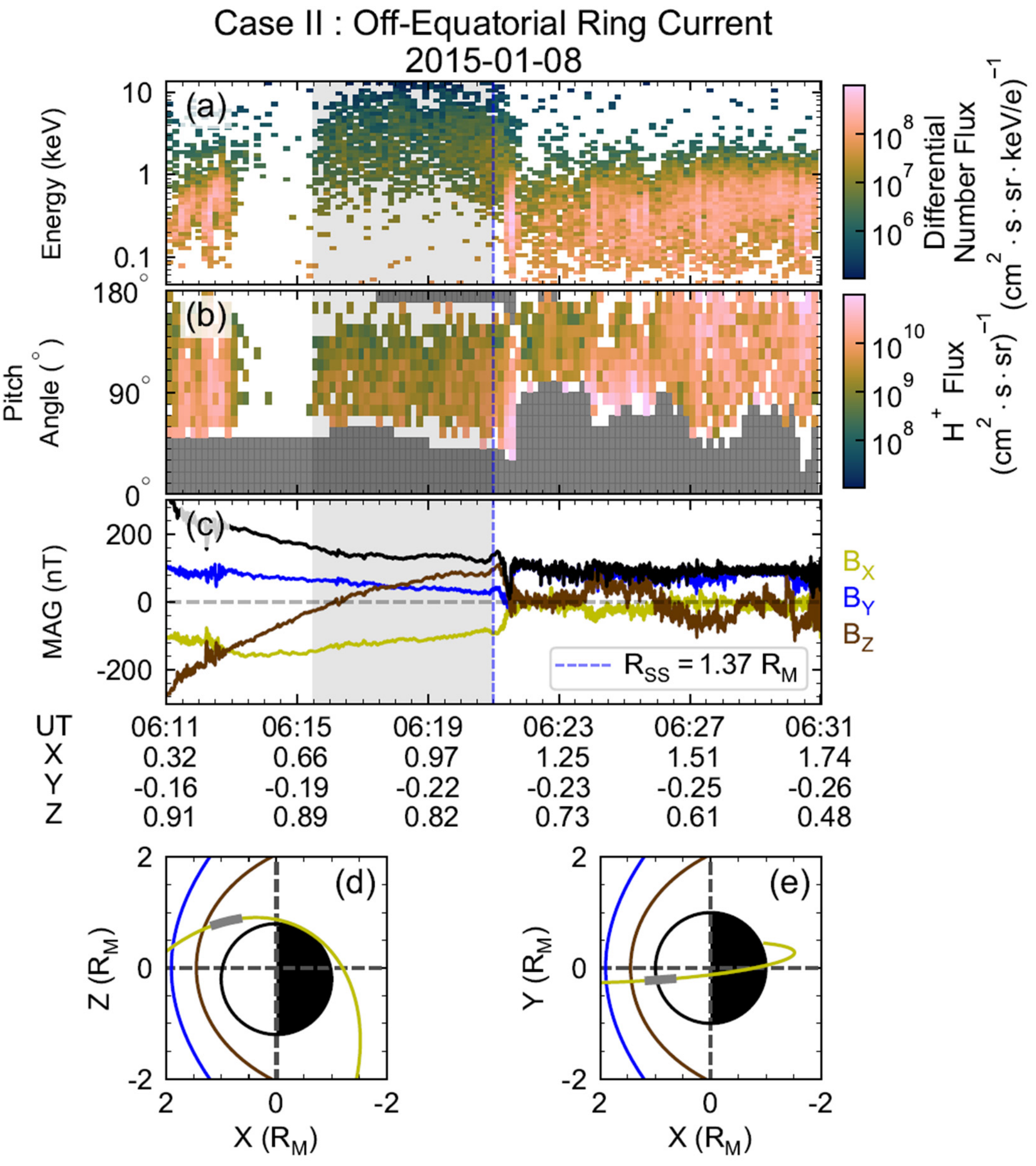

Supplementary Fig. 8. **MESSENGER observations during dayside magnetosphere crossing with potential off-equatorial ring current on Jan 8th, 2015 (Color alternative version of Figure 2).** (a) Energy spectrum of the protons. (b) Corresponding pitch angle distribution, with uncovered pitch angle bins noted by grey grids. (c) Components of the magnetic field (yellow, blue, and brown solid lines represent the B_x, B_y and B_z components, respectively) and strength (solid black line). (d-e) the trajectory of MESSENGER in XZ, XY planes. The thick shaded areas in (a-c) indicate the time periods during which FIPS observed trapped energetic protons. The corresponding spacecraft locations during these intervals are plotted in (d-e) as bold grey lines, and the blue and brown curves show the modelled dayside bow shock and magnetopause that is obtained from the statistical distribution of observed crossing points[12], respectively.

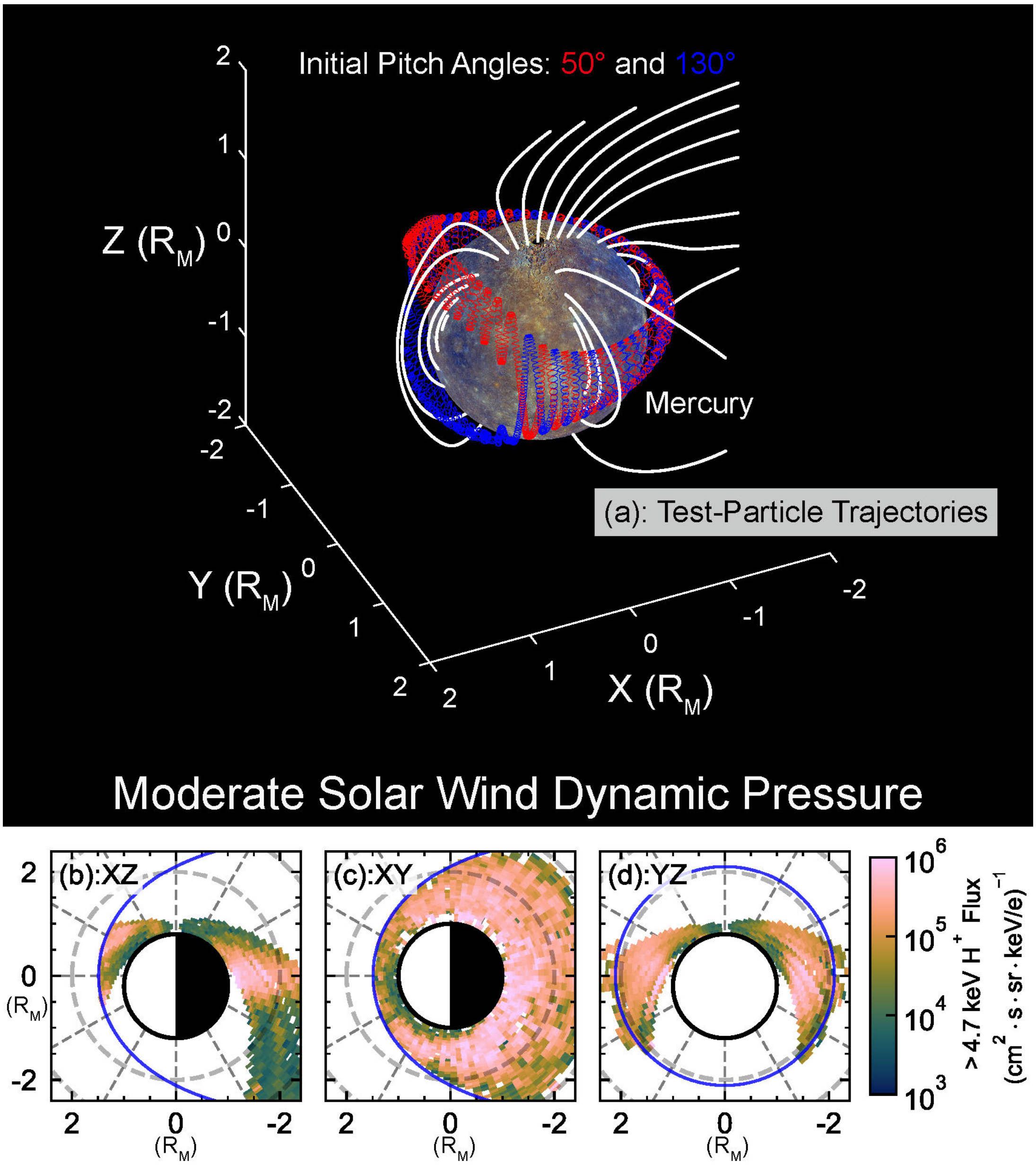

Supplementary Fig. 9. **Comparison of the test-particle simulation and MESSENGER observations of Mercury's off-equatorial ring current (Color alternative version of Figure 3).** (a) 3D view of the trajectories of the 5 keV test protons shown by the red and blue curves with magnetic field lines shown by the white curves. The model parameters r_{Hel} and DI are 0.387 AU and 50, respectively, corresponding to a R_{SS} of 1.41 $\mathrm{R_M}$. (b, c, d) Energetic proton flux distributions based on MESSENGER observations in the day-night (Local Time: 11 h-13 h & 23 h -01 h), geomagnetic equatorial (|*Z*|<0.2 $\mathrm{R_M}$), and dawn-dusk planes (Local Time: 5 h-7 h & 17 h-19 h) under moderate solar wind $\mathrm{p_{dyn}}$ ($1.35\mathrm{R_M} < R_{SS} < 1.49\mathrm{R_M}$).

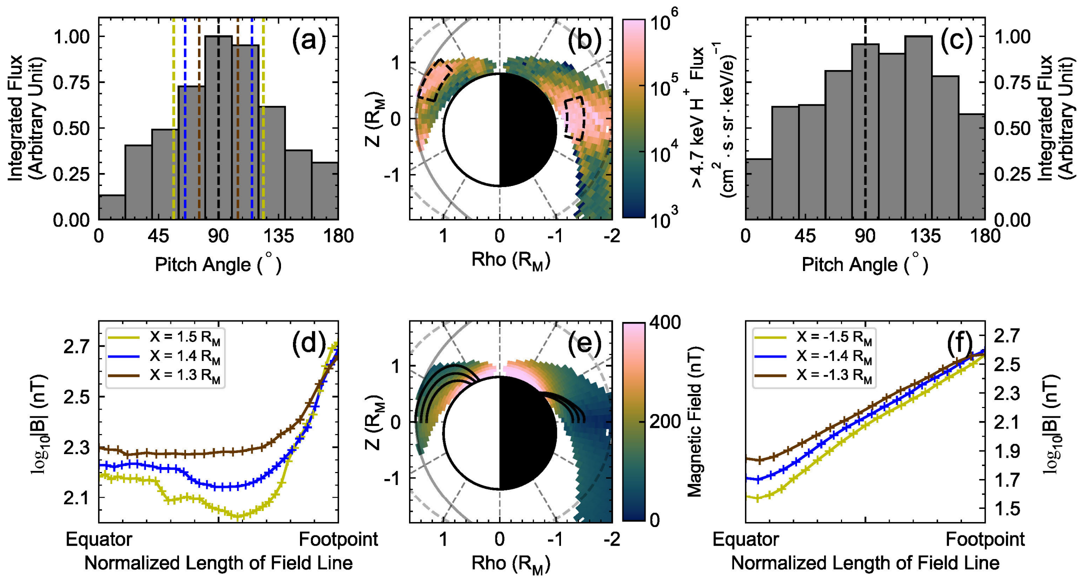

Supplementary Fig. 10. **Pitch angle distributions and field line tracing results (Color alternative version of Figure 4).** (a) The pitch angle distribution of energetic protons in the dayside high latitude magnetosphere (observations inside the area marked by the black box on the dayside in Panel b). (b) The spatial distribution of the energetic proton flux in the day-night meridian plane (Rho $= X \cdot \sqrt{1 + Y^2/X^2}$), adopted from Fig. 2b. (c) The pitch angle distribution of energetic protons in the nightside equatorial magnetosphere (observations inside the area marked by the black box on the nightside in Panel b). (d) Magnetic field strength variations along the field lines originating from $\mathrm{X} = 1.3, 1.4, 1.5\ \mathrm{R_M}$ ($\mathrm{Y} = \mathrm{Z} = 0$). (e) Observed mean magnetic field strength distribution (colour plots) and magnetic field line tracing results (the overplotted black lines). (f) Magnetic field strength variations along the field lines originating from $\mathrm{X} = -1.3, -1.4, -1.5\ \mathrm{R_M}$ ($\mathrm{Y} = \mathrm{Z} = 0$).

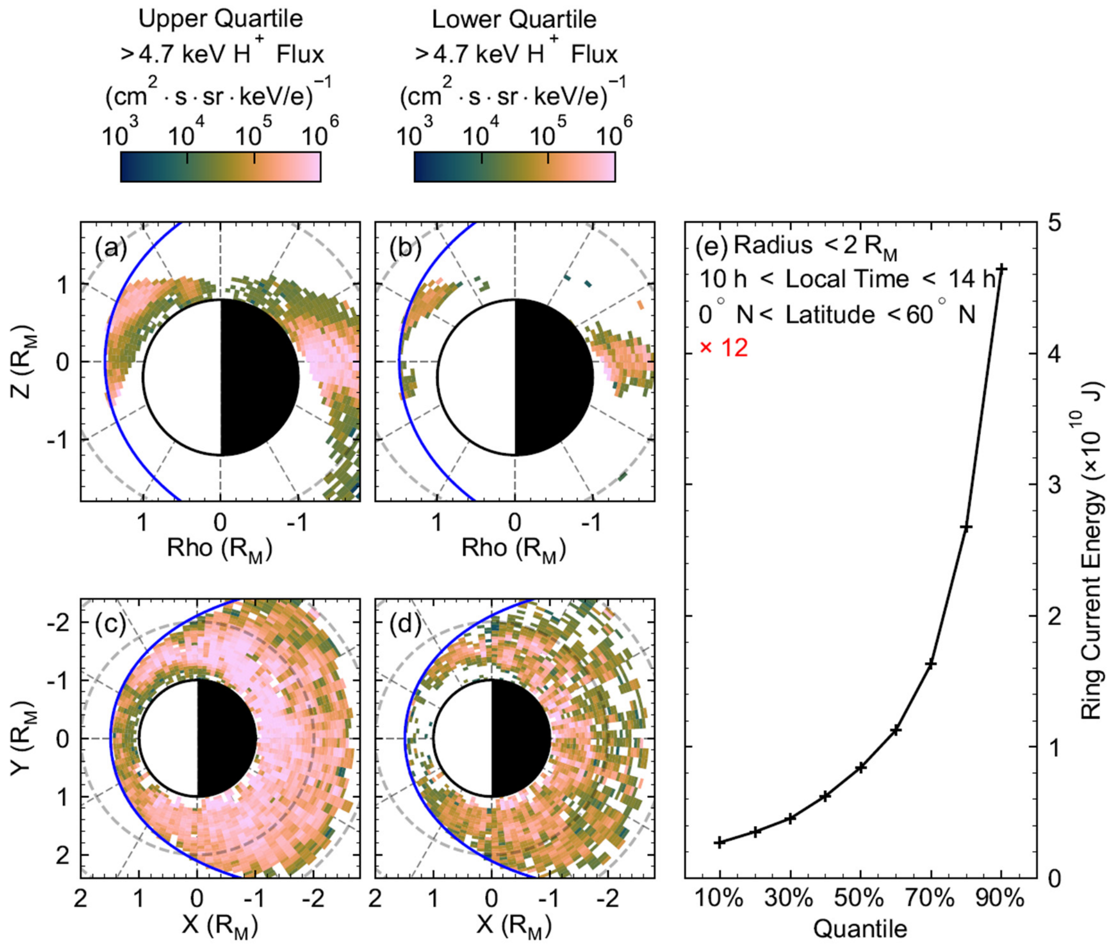

Supplementary Fig. 11. **Ring current variability and its total energy content under moderate solar wind conditions (Color alternative version of Figure 5).** (a, b) Distribution of the upper and lower quartiles of the energetic proton flux in the day-night meridian plane (Rho $= X \cdot \sqrt{1 + Y^2/X^2}$). (c, d) Distribution of the upper and lower quartiles of the energetic proton flux in the equatorial plane. (e) Quantile distribution of the ring current proton's total energy.

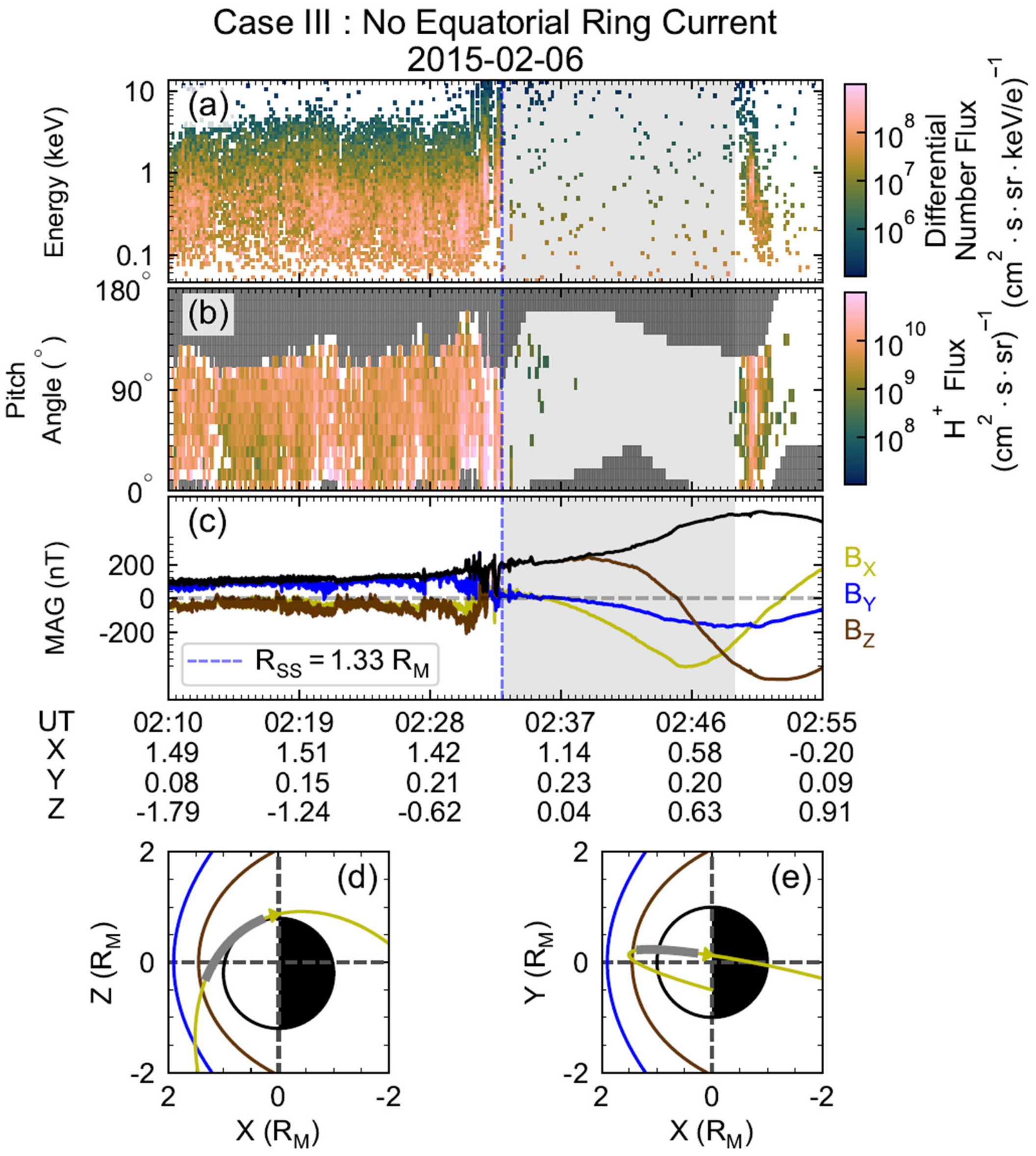

Supplementary Fig. 12. **MESSENGER's observations during dayside magnetosphere crossing without ring current under strong solar wind forcing on Feb 6th, 2015 (Color alternative version of Supplementary Figure 1).** (a) Energy spectrum of the proton. (b) Corresponding pitch angle distribution, with uncovered pitch angle bins noted by grey grids. (c) Magnetic field vectors (red, green, and blue solid lines represent the B_x, B_y and B_z components, respectively) and strength (black solid line). (d, e) the trajectory of MESSENGER in XZ, XY planes. The thick shaded area indicates the time period during which MESSENGER passes through the dayside magnetosphere of Mercury without sensible proton flux enhancement. The corresponding spacecraft locations during this interval are plotted in (d, e) as grey bold lines.

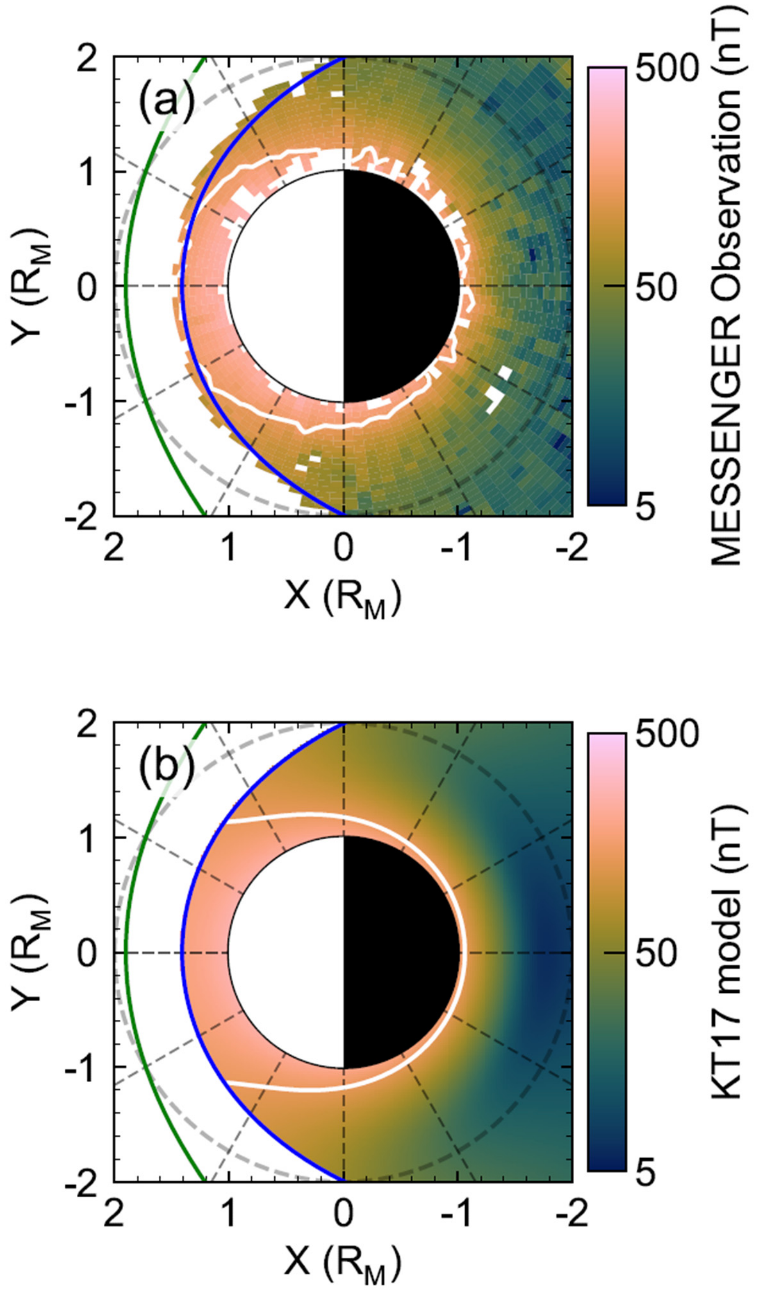

Supplementary Fig. 13. **Magnetic field strength distributions on the equatorial plane (Color alternative version of Supplementary Figure 2).** (a) The observed mean magnetic field around the geomagnetic equator (|*Z*|<0.2 R_M). (b) KT17 magnetic field model ($r_{Hel} = 0.387$ AU, DI $= 50$). Blue and green solid lines represent the modelled magnetopause and bow shock, respectively. The white solid line is the contour of the magnetic field with a strength of 140 nT, close to the magnetic field strength at the mirror point of our initial test particle.

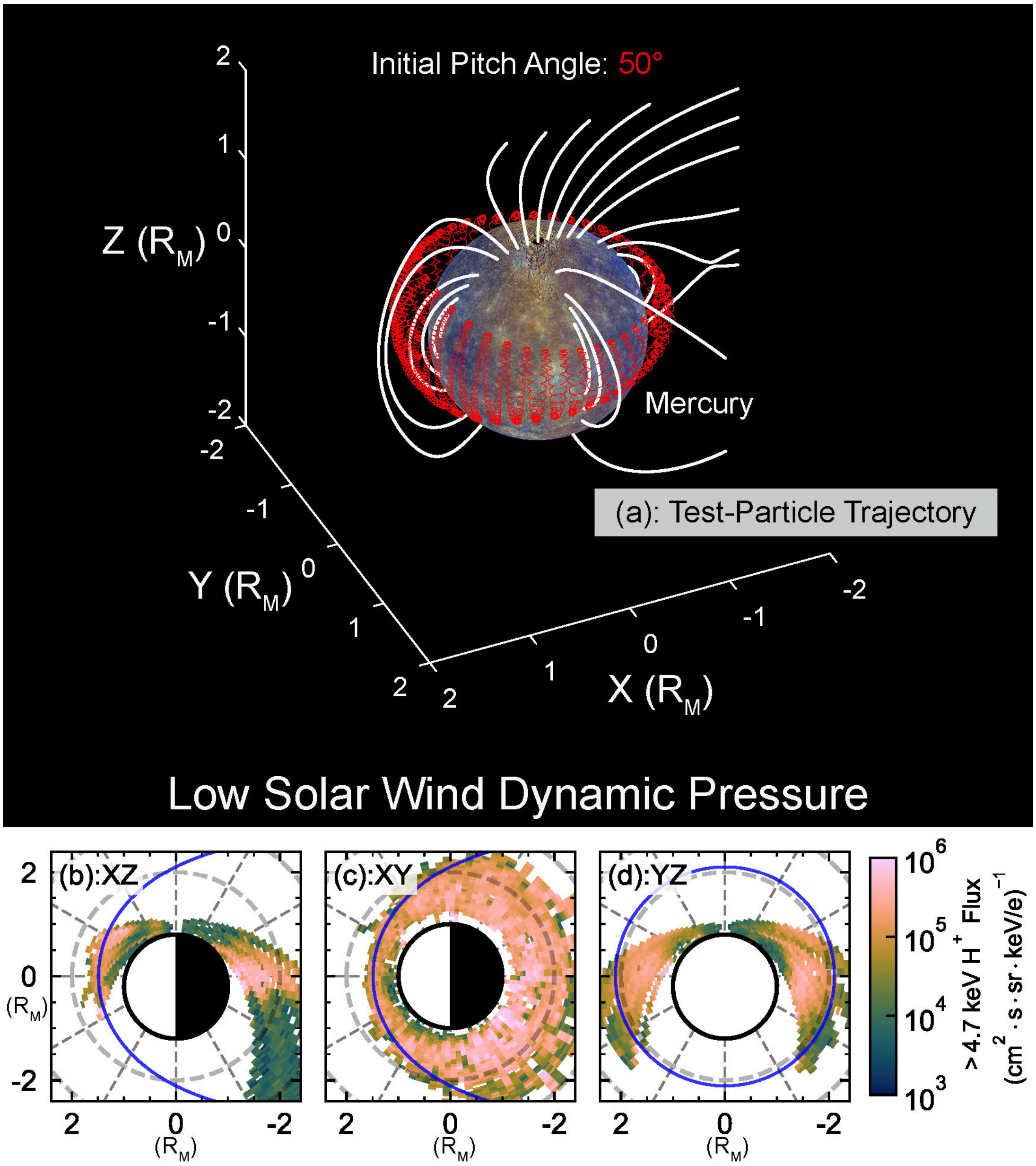

Supplementary Fig. 14. **Comparison of the test-particle simulation and MESSENGER observations of Mercury's equatorial ring current. (Color alternative version of Supplementary Figure 3)** (a) 3D view of the trajectory of the test proton shown by the red curve with magnetic field lines shown by the white curves. The model parameters r_{Hel} and DI are 0.467 AU and 0, respectively, corresponding to a R_{SS} of 1.62 $\mathrm{R_M}$. (b, c, d) Energetic proton flux distributions based on MESSENGER observations on the day-night (local time: 11 h-13 h & 23 h -01 h), geomagnetic equatorial ($|Z| < 0.2$ $\mathrm{R_M}$), and dawn-dusk planes (local time: 5 h-7 h & 17 h-19 h) under low solar wind p_{dyn} ($1.49\mathrm{R_M} < R_{SS}$).

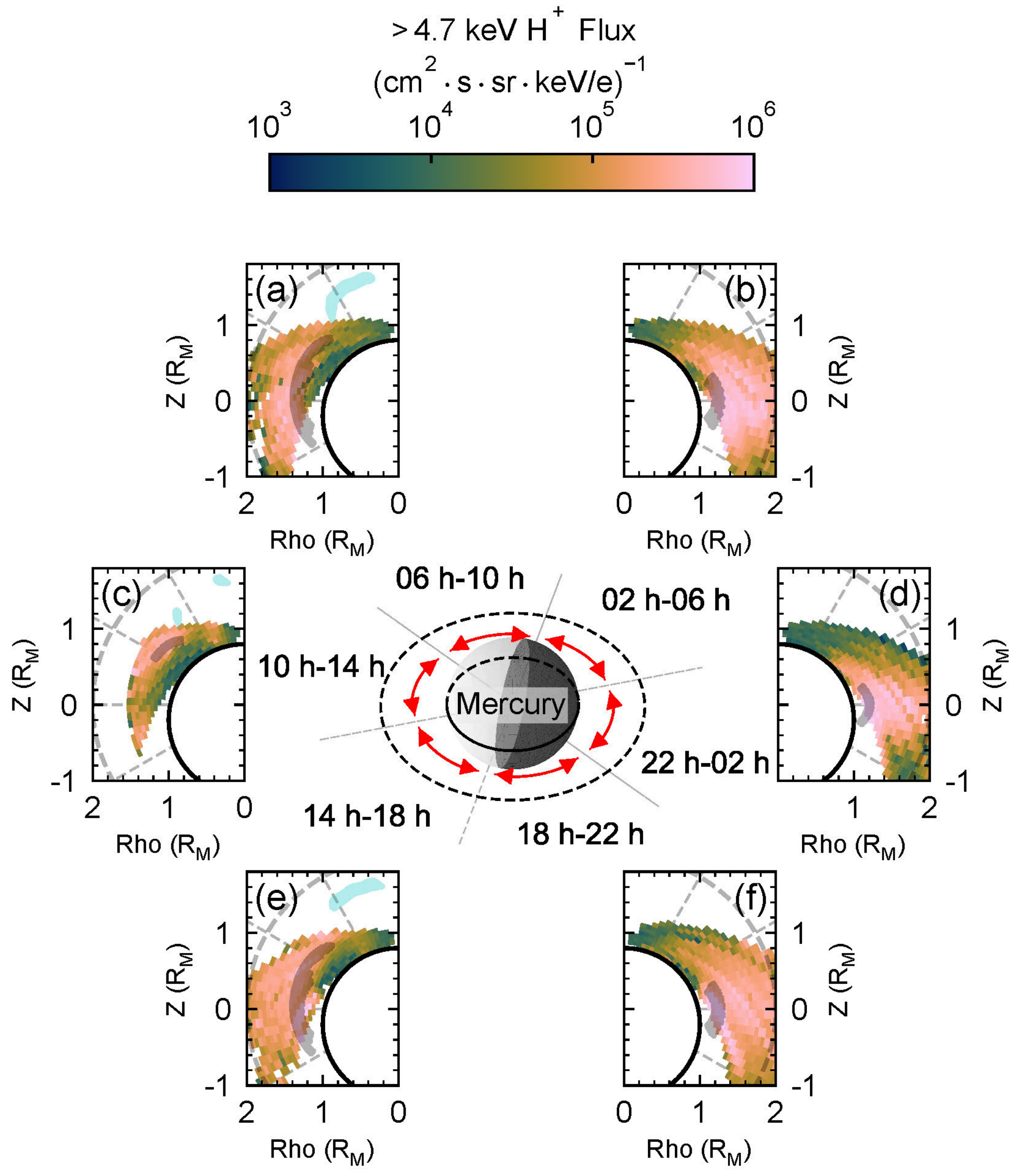

141

142 Supplementary Fig. 15. **Comparison of the test particle trajectories and MESSENGER observations**
143 **(Color alternative version of Supplementary Figure 5).** (a-f) Statistical distributions of energetic
144 proton flux in the meridian plane within different local time ranges. The ring current particle trajectory
145 is overplotted as a grey area. The cusp trapped proton trajectory is overplotted as a cyan area for
146 comparison.

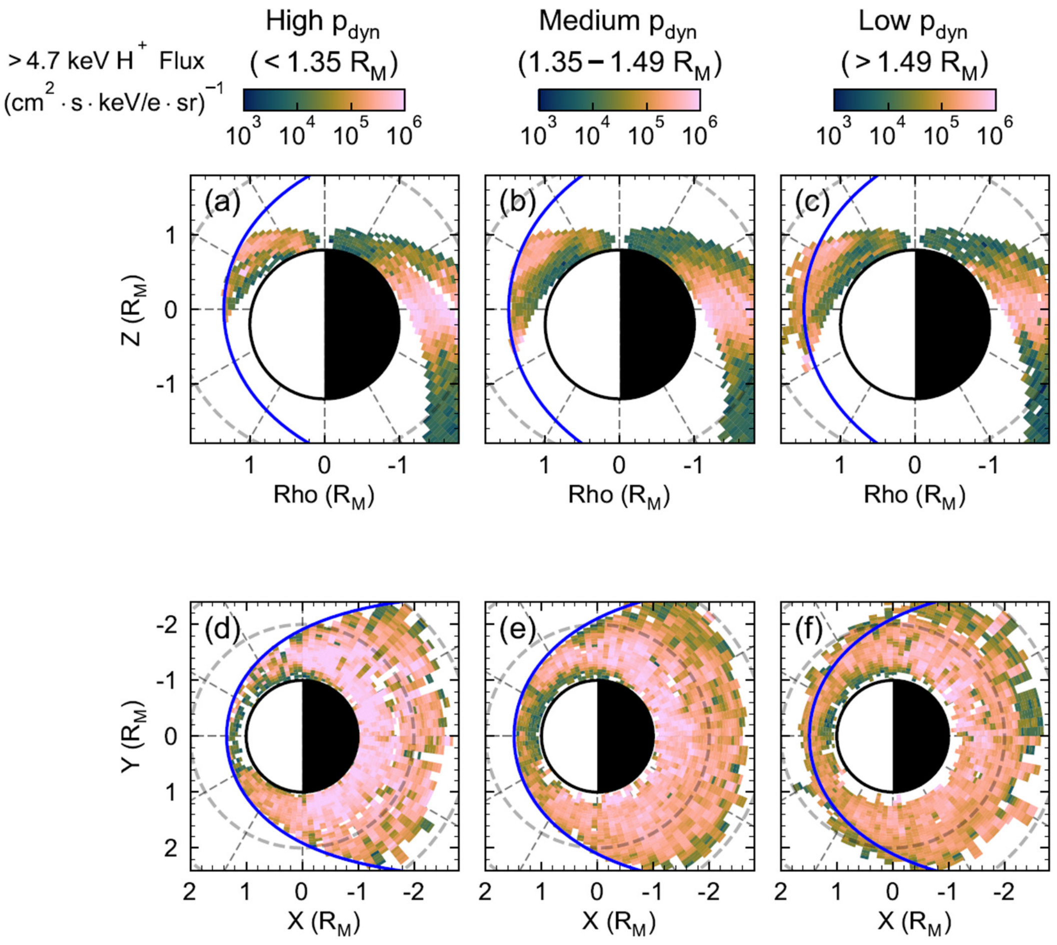

Supplementary Fig. 16. **Distributions of energetic proton fluxes in units of $\mathbf{cm^{-2} \cdot s^{-1} \cdot sr^{-1} \cdot (keV/e)^{-1}}$ under different solar wind forcing conditions (Color alternative version of Supplementary Figure 6).** (a-c) Energetic proton flux distributions in the day-night plane (local time: 11 h-13 h & 23 h -01 h) under high, moderate, and low solar wind forcing conditions. (d-f) Energetic proton flux distributions in the geomagnetic equatorial planes ($|Z|<0.2$ R_M) under high, moderate, and low solar wind forcing conditions.

Supplementary References

1. Crameri, F. Scientific colour maps (7.0.1). Zenodo. https://doi.org/10.5281/zenodo.5501399 (2021).
